# High-fidelity dispersive spin sensing in a tunable unit cell of silicon MOS quantum dots

**DOI:** 10.1038/s44460-026-00084-6

**Published:** 2026-07-07

**Authors:** Constance Lainé, Giovanni A. Oakes, Virginia Ciriano-Tejel, Jacob F. Chittock-Wood, Lorenzo Peri, Michael A. Fogarty, Sofia M. Patomäki, Stefan Kubicek, David F. Wise, Ross C. C. Leon, M. Fernando Gonzalez-Zalba, John J. L. Morton

**Affiliations:** 1 Quantum Motion, London, United Kingdom; 2 London Centre for Nanotechnology, UCL, London, UK; 3 Cavendish Laboratory, University of Cambridge, Cambridge, UK; 4 IMEC, Leuven, Belgium; 5 CIC nanoGune Consolider, Donostia-San Sebastian, Spain; 6 IKERBASQUE, Basque Foundation for Science, Bilbao, Spain; 7 Present Address: Center for Emergent Matter Science, RIKEN, Saitama, Japan

**Keywords:** Nanosensors, Qubits, Quantum dots, Quantum dots, Characterization and analytical techniques

## Abstract

Metal–oxide–semiconductor (MOS) technology offers a promising route to scalable quantum computing based on spin qubits, but large-scale architectures require compact and sensitive sensors that preserve qubit connectivity and remain compatible with industrial fabrication. Here we demonstrate a dispersive spin–qubit sensor, a single-electron box (SEB), integrated within a bilinear unit cell of planar MOS quantum dots fabricated using an industrial 300-mm wafer process. Independent gate control of the SEB and double-quantum-dot tunnel rates enables optimization of the sensor, achieving state-of-the-art dispersive readout fidelities of 99.92% in 340 μs and 99% in 20 μs. We also develop a hidden Markov model of the two-electron spin dynamics, allowing a more accurate determination of the measurement outcome and corresponding fidelity. Our results show that the compactness and versatility of SEB-based charge sensing can be realized without compromising sensitivity, providing a scalable pathway for future MOS spin–qubit architectures.

## Abstract (web summary)

A compact, industrially fabricated single electron box sensor enables state-of-the-art high-fidelity dispersive spin readout within a tunable silicon MOS quantum dot unit cell.

## Main

An essential component within a scalable quantum processor unit (QPU) is a qubit measurement device that combines high readout fidelity with a minimized physical footprint^1^. For semiconductor spin qubits, readout is usually performed by mapping the spin to a charge state, which is detected using a nearby charge sensor such as the single-electron transistor (SET)^2–4^, which has set the standard for fast high-fidelity readout (see Extended Data Table 1 and Extended Data Fig. 1 in Supplementary Note A). Recently, a more compact type of charge sensor has emerged: the radiofrequency (RF) single-electron box (SEB), which consists of a single charge reservoir coupled to a quantum dot (QD), whose impedance is measured via RF reflectometry^5–8^. In addition to being more compact, SEBs have been predicted to offer sensitivities approaching those of SETs, assuming suitable resonator and device optimization^9^.

SEB sensors have been used in diverse semiconductor systems^10,11^ and have potential applications in other QPU modalities such as Majorana^12^ or electrons on helium or neon^13^. Beyond charge sensing, SEBs also enable a broad range of electronic applications, including nanoscale thermometry^14,15^, energy spectroscopy^16^, frequency-domain signal processing (such as quantum-limited parametric amplification or frequency mixing^17,18^) and the study of many-body fermionic systems^19^. Developing SEBs open new opportunities for high-sensitivity, low-power electronics as well as high-fidelity readout in qubit architectures with enhanced connectivity.

For quantum computing, semiconductor spin qubits present the advantage of potential compatibility with high-yield fabrication processes of the semiconductor industry^20^, particularly for architectures based on existing transistor technology, such as the planar silicon metal–oxide–semiconductor (MOS) technology^21^. MOS quantum dots have enabled high-fidelity single- and two-qubit gates^22,23^, where readout is usually achieved using SETs, taking advantage of their high sensitivity despite their larger footprint.

In this Article we demonstrate an SEB in a planar MOS process and we study whether this type of charge sensor can offer readout speed and fidelity comparable to SETs, while offering more scalable architectures. We use a QPU unit cell comprising one row of QDs hosting spin qubits, and a parallel row including an SEB. Our approach is to leverage the electrical tunability afforded by multiple overlapping gates, available from the planar MOS fabrication. By adjusting the potential of barrier gates between adjacent QDs, we optimize the signal-to-noise ratio (SNR) and readout fidelity.

Next, we introduce a hidden Markov model (HMM) that considers the full readout dynamics and accounts for the three possible states of the double QD (DQD) spin system. This approach improves fidelity and classification confidence over the traditional methods that simply consider two states. Together, these device-level and analytical advances allow us to optimize spin relaxation rates for maximum fidelity in parity readout, which we find exceeds 99.9%. This use of sensitive charge detection and HMM time-series analysis provides a general framework for ultrasensitive multi-level charge detection, with potential applications in a broad range of systems in which signals from different physical states overlap. Examples include cryogenic photon detection^24^, spin measurement in carbon nanotubes^25^ and qubit readout in superconducting circuits where HMM has already been explored to improve qubit classification^26^.

## QPU unit cell with a scalable sensor

**Fig. 1:**
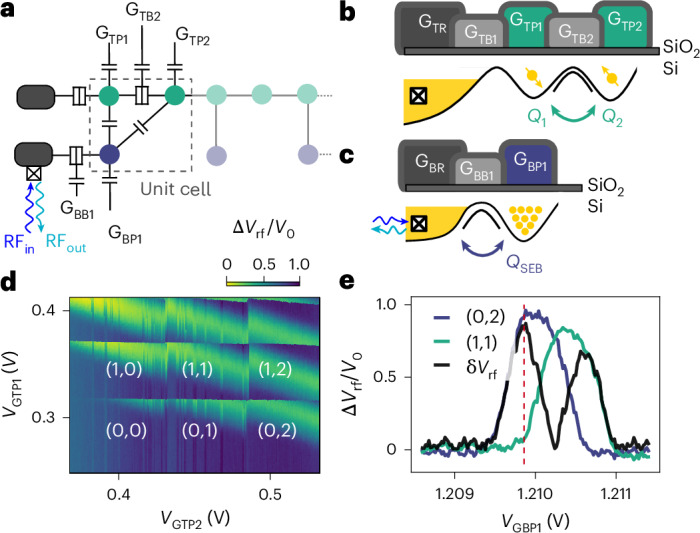
Unit cell for quantum computation in planar silicon MOS with an array of sensors and qubits. **a**, Schematic of the device, showcasing a scalable architecture for a silicon QPU with a repeating unit cell containing one sensor dot (purple) and two qubit dots (blue). QDs are represented by a circle, and tunnel barriers by a capacitance in parallel to a resistance. The dots and barriers are capacitively coupled to gates. **b**, Cross-sectional view of the top row, tuned as a DQD with charges of *Q*_1_ and *Q*_2_, respectively, under plunger gates G_TP1_ and G_TP2_. The barrier gate TB2 regulates the tunnel coupling between dots. **c**, Cross-sectional view of the bottom row, with the sensor SEB tuned in the many-electron regime. A RF tone is sent to the ohmic contact of the reservoir gate for reflectometry. Barrier gate BB1 regulates tunnel coupling between the dot and reservoir. **d**, Charge stability diagram of the qubit dots with electron occupation down to the first electrons. **e**, Coulomb peak of the SEB when the qubit array is in the (1, 1) or (0, 2) electron occupation. We maximize the difference in signal ${\rm{\delta }}{V}_{\mathrm{rf}}=|\Delta {V}_{\mathrm{rf}}^{(11)}-\Delta {V}_{\mathrm{rf}}^{(02)}|/{V}_{0}$ by operating at the red dashed line.

We consider a QPU architecture consisting of a bilinear QD array, as shown in Fig. 1a. In this design, the sensor is not co-linear with the qubits, allowing lateral scaling without reducing sensitivity, and is usable as a readout component in fault-tolerant architectures based on bilinear arrays^27^. The device studied here corresponds to a unit cell within the larger array of Fig. 1a. It is fabricated using an industrial-grade 300-mm-wafer, planar MOS ^nat^Si process^28^. The top row features a DQD with a tunable barrier gate, and the bottom row contains the SEB dot, tunnel-coupled to an electron reservoir extending from an ohmic implanted region via an accumulation gate (Fig. 1b,c). The ohmic contact is connected to a superconducting *LC* resonator for readout (see Extended Data Table 2 and Extended Data Fig. 2 in Supplementary Note B).

Charge sensing is performed using the SEB dot in the many-electron regime, monitoring a Coulomb blockade peak produced by an electron cyclically tunnelling between the dot and the reservoir. This enables the top row DQD to be sensed in the single-electron regime, as evidenced by the charge stability diagram in Fig. 1d. To perform Pauli spin blockade (PSB), we focus on the region of the interdot charge transition (ICT) between the (1, 1) and (0, 2) charge states of the DQD, and adjust the SEB plunger gate to the point of maximal contrast (Fig. 1e).

**Fig. 2:**
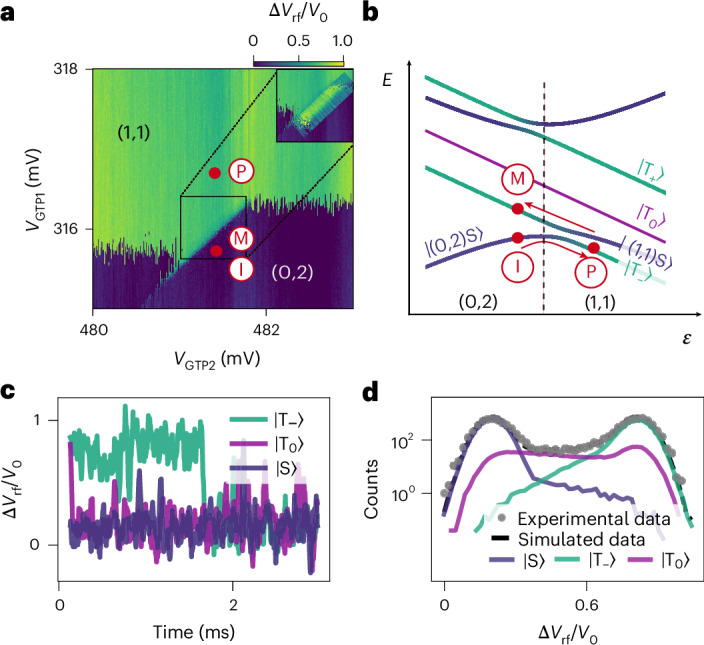
Spin readout protocol. **a**, Charge stability diagram of the qubit array around the (1, 1)–(0, 2) ICT, with the readout voltage pulse sequence overlaid. The sequence consists of three steps: ‘I’ for initialization to |S(0, 2)〉, ‘P’ for plunge to (1, 1), and ‘M’ for measurement. The sequence is detailed in Supplementary Note C. The PSB window with the persistence of (1, 1) signal is shown in the inset. **b**, Energy levels for a DQD with two electrons, as a function of detuning *ε*, the potential difference between the two QDs. At a finite magnetic field, the Zeeman energy splits the triplet states, and the singlet is coupled to the polarized triplet via spin–orbit coupling. The pulse sequence points are also overlaid. **c**, Single-shot traces for |S〉, |T_−_〉 and |T_0_〉. **d**, Histogram of 10,000 single-shot traces averaged over the first *t*_read_ = 204 μs after correcting for sensor drift (see Extended Data Fig. 5) (scatter plot), alongside histograms of |S〉, |T_−_〉, |T_0_〉 and their sum (black line) for data simulated via the HMM (see Extended Data Fig. 6 in Supplementary Note F). The state probabilities are $p(|{\rm{S}}\rangle )=p(|{{\rm{T}}}_{0}\rangle )=0.25$ and $p(|{{\rm{T}}}_{-}\rangle )=0.5$. The dataset reveals decay times ${\varGamma }_{{{\rm{T}}}_{-}}^{-1}=290\,{\rm{ms}}$ and ${\varGamma }_{{{\rm{T}}}_{0}}^{-1}=0.170\,{\rm{ms}}$. The simulated data include the effect of a two-level fluctuator for a better match with the experimental data.

We perform PSB at the ICT shown in Fig. 2a under a static in-plane magnetic field (*B*_0_ = 100 mT) which lifts the degeneracy between triplet states, producing the energy levels in Fig. 2b. A pulse sequence (laid on Fig. 2a,b) prepares a superposition of the three possible two-electron states $|{\rm{S}}\rangle =\frac{1}{\sqrt{2}}(|\uparrow \downarrow \rangle -|\downarrow \uparrow \rangle )$, $|{{\rm{T}}}_{0}\rangle =\frac{1}{\sqrt{2}}(|\uparrow \downarrow \rangle +|\downarrow \uparrow \rangle )$ or $|{{\rm{T}}}_{-}\rangle =|\downarrow \downarrow \rangle$ by ramping semi-adiabatically across the |S〉– |T_−_〉 anticrossing (see Extended Data Fig. 3 in Supplementary Note C). At the measurement point in the (0, 2) region, singlet state |S〉 rapidly transitions from (1, 1) to (0, 2), whereas triplets |T_−_〉 and |T_0_〉 are blocked in (1, 1) until they relax to the singlet (0, 2) ground state at the respective rates ${\varGamma }_{{{\rm{T}}}_{-}}$ and ${\varGamma }_{{{\rm{T}}}_{0}}$. The blockade is visible in the stability diagram as persistence of the (1, 1) signal near the ICT (inset, Fig. 2a). PSB is lifted at larger detuning when an excited triplet (0, 2) state becomes accessible. From the width of the readout window and the estimated ICT lever arm of *α*_ICT_ = 0.17 (see Extended Data Fig. 4 in Supplementary Note D), we extract an excited-state energy in dot TP2 of 25 μeV, probably due to a low-lying excited valley state^29,30^.

PSB was further studied by monitoring the signal in the time domain immediately after pulsing to the measurement point, (three typical single-shot traces are shown in Fig. 2c). Signals corresponding to the (0, 2) configuration can be ascribed to |S〉, while other traces that show the (1, 1) configuration for a finite time before relaxation are assigned to one of the two triplets, |T_−_〉 or |T_0_〉, distinguishable from their relaxation rates ${\varGamma }_{{{\rm{T}}}_{-}}\ll {\varGamma }_{{{\rm{T}}}_{0}}$ (Supplementary Note C).

This Article considers two approaches to classify the qubit state from a single-shot trace. The first, and commonly used, method (referred to as the ‘threshold method’) averages the time-domain signal over some window *t*_read_ and compares the average to a predefined threshold. The second method (called the ‘HMM method’) uses a Gaussian HMM^31,32^ to infer the state using all data points within the time window (Methods). The threshold method distinguishes two states based on the integration time *t*_read_ and the relevant relaxation times^33^. Setting ${t}_{{\rm{read}}}\ll {\varGamma }_{{{\rm{T}}}_{0}}^{-1}$ enables singlet–triplet readout (discriminating |S〉 from (|T_−_〉, |T_0_〉)), whereas setting ${\varGamma }_{{{\rm{T}}}_{0}}^{-1}\ll {t}_{{\rm{read}}}\ll {\varGamma }_{{{\rm{T}}}_{-}}^{-1}$ achieves spin parity readout (discriminating even-parity (|T_−_〉) from odd-parity (|S〉, |T_0_〉) states). The HMM can instead distinguish, in a single-shot trace, between the three states |S〉, |T_0_〉 and |T_−_〉, going beyond the two-readout basis.

## Results

### Accurate modelling of three-state distribution

In calculating qubit readout fidelity, a common challenge is to distinguish contributions from state preparation and measurement (SPAM) errors, often measured altogether by comprehensive tomography techniques^34^ or repetitive measurements^35^. Alternatively, the readout error alone can be estimated by modelling the measurement distribution under some assumptions on the physical system. In this case, the reported fidelity depends highly on the validity of the model and the quality of the fit to the experimental data^36^.

We first assess readout fidelity using the threshold method and consider the expected distribution of the averaged value of a single-shot trace. Figure 2d presents a histogram of 10,000 time-averaged single-shot traces (after compensating for sensor drift; Supplementary Note E). A common approach is to model these data using a bimodal probability density distribution^37^, assuming that only two spin states are involved: |S〉 and one of the triplet states (for example, |T_−_〉 for parity readout). The readout fidelity and optimal threshold are then calculated from this modelled distribution (Methods). In the presence of Gaussian noise, the readout fidelity is related to the voltage SNR of the time-dependent spin readout signal^26^:

1 $${F}_{{\rm{m}}}^{\ast }=\frac{1}{2}\left[1+\mathrm{erf}\left(\frac{\mathrm{SNR}({t}_{\mathrm{read}})}{2\sqrt{2}}\right)\exp \left(-\frac{\varGamma {t}_{\mathrm{read}}}{2}\right)\right]$$

where, *Γ* is the relaxation rate of the relevant triplet.

A clear limitation of this two-state model is its failure to account for additional states of the two-spin system, which becomes particularly important when ${t}_{{\rm{read}}}\sim {\Gamma }_{{{\rm{T}}}_{0}}^{-1}$ and ${t}_{{\rm{read}}}\ll {\varGamma }_{{{\rm{T}}}_{-}}^{-1}$. In this regime, the contribution from the |T_0_〉 state appears at the intersection of the peaks from |S〉 and |T_−_〉 (see also Fig. 7 in Methods) and is not captured by the two-state model. This introduces additional measurement errors, such that readout fidelity is overestimated, as we shall see.

To improve the model accuracy, we simulate the probability distribution using single-shot traces generated by a HMM that includes three hidden states (|S〉, |T_0_〉 and |T_−_〉) and noise from a two-level fluctuator (TLF) (Methods). This produces a simulated histogram that matches well with the experimental data, as seen in Fig. 2d. In the following, we compare measures of parity readout fidelity using either the two-state model or the HMM for data simulation, in both cases distinguishing in the parity basis with the threshold method (see Table 1 in Methods). This highlights the regime where a two-state model is insufficient to estimate readout fidelity. We use ${F}_{{\rm{m}}}^{\ast }$ to refer to the (typically overestimated) value from the two-state model and *F*_m_ for the value obtained using the HMM.

### Maximizing the charge-readout signal

Regardless of the method used to model single-shot data, improving the charge sensor SNR is crucial to enhance spin readout fidelity and speed, as evidenced by equation (1). For an SEB, the signal amplitude (and thus SNR) is related to the capacitance change Δ*C*_DRT_ between blocked and fully degenerate charge states of the SEB due to a dot-to-reservoir transition (DRT) (Supplementary Note G). Experimentally, the maximum signal is the amplitude of the Coulomb peak $\Delta {V}_{{\rm{rf}}}^{\rm max}$. However, for charge sensing, the relevant signal arises from the fractional capacitance change caused by a charge transition (for example, between (1, 1) and (0, 2) in the DQD) and is given by the contrast δ*V*_rf_. These quantities are related by ${\rm{\delta }}{V}_{\mathrm{rf}}=\eta \Delta {V}_{\mathrm{rf}}^{\rm max}$ where *η* is a dimensionless parameter between 0 and 1: *η* = 1 when the (0, 2) and (1, 1) peaks are well separated relative to the SEB linewidth, and *η* = 0 when they fully overlap. Charge readout therefore requires maximizing the product *η*Δ*C*_DRT_, where Δ*C*_DRT_ follows^38^

2 $$\Delta {C}_{\mathrm{DRT}}\simeq \frac{4{\alpha }^{2}{e}^{2}}{3{k}_{{\rm{B}}}{T}_{{\rm{e}}}}\mathop{\underbrace{\frac{1}{1+{({f}_{\mathrm{rf}}/\gamma )}^{2}}}}\limits_{({\rm{i}})}\mathop{\underbrace{\frac{1}{1+h\gamma /{k}_{{\rm{B}}}{T}_{{\rm{e}}}}}}\limits_{(\mathrm{ii})}$$

where *α* is the lever arm, *e* is the electron charge, *k*_B_ is Boltzmann’s constant, *h* is Planck’s constant, *T*_e_ is the electron temperature, *f*_rf_ is the RF frequency and *γ* is the DRT tunnel rate controlled by the barrier gate voltage.

Equation (2) reveals two competing terms: as *γ* increases, term (i) increases, as electron tunnelling during a RF cycle becomes more likely, until term (ii) decreases as the charge transition becomes lifetime-broadened and reduces the probability of tunnelling. A sweet spot is found when *γ* ≫ *f*_rf_—here 570 MHz—but still *γ* < *k*_B_*T*_e_ where thermal broadening occurs (1.87 GHz for our device where *T*_e_ = 90 mK) (Supplementary Note D).

**Fig. 3:**
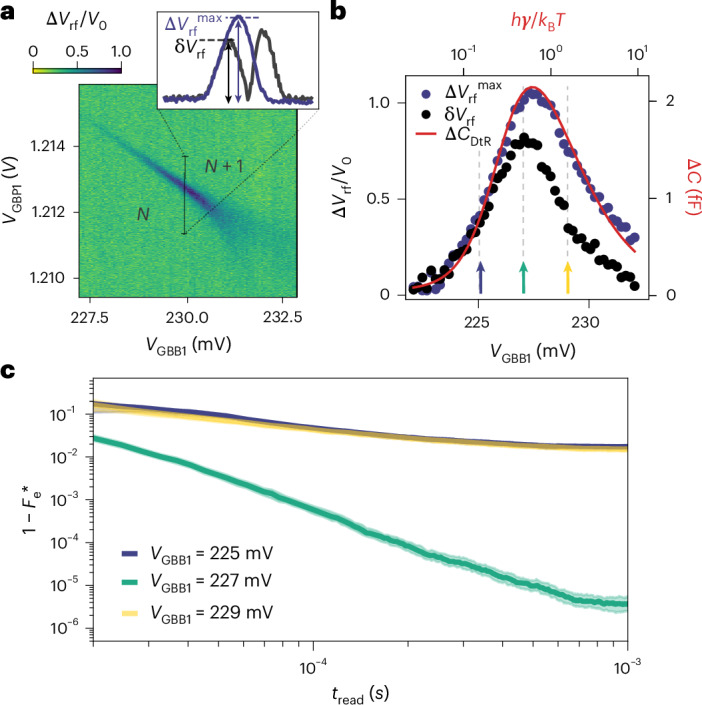
Tuning charge-readout fidelity via the SEB barrier gate. **a**, Stability diagram of the sensor with the plunger BP1 and barrier gate BB1, showing broadening of the peak at large *V*_GBB1_ from the enhanced lifetime. The peak separates regions of different electron numbers *N* and *N* + 1, where *N* ≃ 15. This map is taken at lower power than the rest of the panels to highlight the effect of lifetime broadening, explaining the apparent shift in optimal BB1 voltage (Supplementary Note D). The inset shows the SEB peaks from Fig. 1c, where we define $\Delta {V}_{{\rm{rf}}}^{max}$ as the height of the SEB peak (purple) and δ*V*_rf_ as the contrast between the two charge states (black). **b**, Lifetime broadening of the SEB peak (black-labelled axes), showing the SEB peak height is reduced at *γ* larger than *k*_B_*T*. We superimpose a plot of Δ*C*_DRT_ as a function of tunnel rate calculated from equation (2) (red-labelled axes), with *f*_rf_ = 576 MHz, the lever arm and electron temperature from Supplementary Note D, and tunnel rate *γ* ranging from 0.05 to 19 GHz, assuming *V*_GBP1_ exponentially modulates tunnel rate *γ*. **c**, Electrical fidelity for three different SEB barrier gate voltages, indicated by arrows in **b**. The electrical fidelity is calculated using the threshold method and bimodal fitting. The optimal point (green) corresponds to *γ* = 1.1 GHz. The yellow point is in the lifetime-broadened regime, and the purple point is in the slow-tunnelling regime. In **c**, the error bands (when visible) approximate to ±1*σ* from the fit (Methods).

Both contributions are observed in Fig. 3a, where the charge sensor signal is measured against the barrier gate. The peak height $\Delta {V}_{{\rm{rf}}}^{\rm max}$ initially increases before reducing from lifetime broadening. This behaviour is clearer in Fig. 3b, where we superimpose on the data the calculated change in capacitance Δ*C*_DRT_ against the tunnel rate *γ* calculated from equation (2), assuming *γ* depends exponentially on the barrier voltage. The model agrees well with the data and yields a tunnel rate near the maximum signal at *γ* = 1.1 GHz.

To optimize charge readout, we also examine the contrast δ*V*_rf_ in Fig. 3b, which includes both *η* and $\Delta {V}_{{\rm{rf}}}^{\max }$. The contrast reaches ~80% of the maximum visibility, indicating good coupling between the charge sensor and DQD. We also observe a slight shift in the peak of δ*V*_rf_ compared to the maximum visibility $\Delta {V}_{{\rm{rf}}}^{\rm max}$, because *V*_GBB1_ affects not only the amplitude of the peak but also *η* through changes in the capacitive coupling between the SEB and the DQD. At larger barrier gate bias, lifetime broadening reduces *η* and therefore δ*V*_rf_ more strongly than Δ*C*_DRT_.

The impact of the SEB barrier gate on readout errors is visible when calculating the electrical fidelity ${F}_{{\rm{e}}}^{\ast }$. This fidelity is obtained from the two-state model by neglecting spin-to-charge conversion losses (that is, *Γ* in equation (1) is set to 0 and ${F}_{{\rm{e}}}^{\ast }$ does not include triplet relaxation; Methods) and thus provides an upper bound on spin fidelity. In Fig. 3c, we notice how ${F}_{{\rm{e}}}^{\ast }$ increases with readout time (expected under Gaussian noise when the SNR is proportional to $\sqrt{{t}_{{\rm{read}}}}$) until it plateaus, indicating the onset of 1/*f* charge noise^39^ (see Extended Data Fig. 7 in Supplementary Note H). We also show the improvement in electrical fidelity at the optimal barrier gate voltage and thus DRT tunnel rate. This illustrates the importance of tuning the barrier gate of the SEB to maximize the bounds on qubit readout fidelity.

### Optimizing relaxation for parity readout

**Fig. 4:**
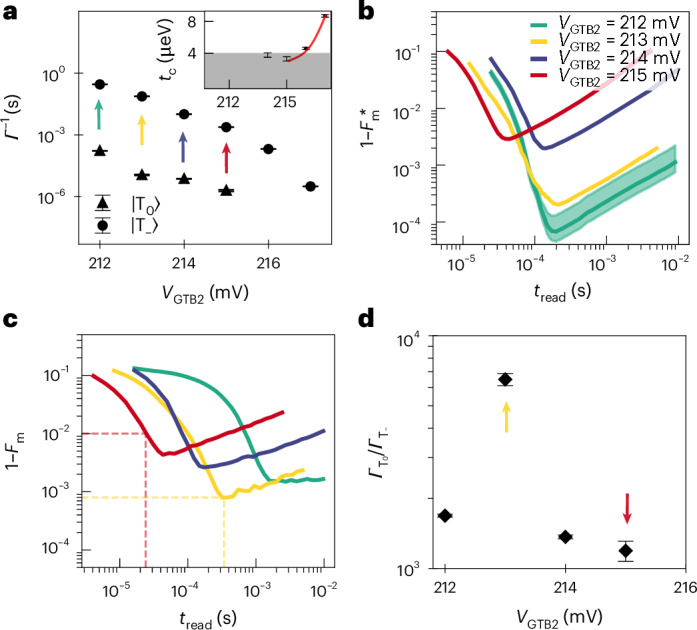
Tuning the triplet relaxation time and spin parity readout fidelity using the threshold method via a qubit barrier gate. **a**, ${\varGamma }_{{{\rm{T}}}_{-}}^{-1}$ and ${\varGamma }_{{{\rm{T}}}_{0}}^{-1}$ show an exponential dependency on barrier gate voltage. For *V*_GTB2_ < 212 mV, the dots become too weakly coupled, with an ICT size smaller than voltage resolution, preventing measurement of the PSB. For *V*_GTB2_ > 217 mV, the two QDs begin to merge, and the relaxation times of both triplets are below the measurement bandwidth of 3.3 μs (Supplementary Note J). Inset: tuning of tunnel coupling with barrier gate voltage (see Extended Data Fig. 8 in Supplementary Note I), where the linewidth saturates at 4 μeV for *V*_GTB2_ < 215 mV (grey area) due to thermal broadening with a qubit electron temperature of *T*_e_ = 40 mK. **b**,**c**, Spin parity fidelity varies as a function of the qubit barrier gate. Fidelity is calculated using either the threshold method and simulating the spin distribution via the simple two-state model (**b**) or the HMM, which accounts for a three-state distribution and TLF (**c**). In both cases, the initial distribution is fixed to $p(|{\rm{S}}\rangle )=p(|{{\rm{T}}}_{0}\rangle )=0.25$ and $p(|{{\rm{T}}}_{-}\rangle )=0.5$ corresponding to 50/50 odd–even parity distribution that does not bias the overall fidelity towards a certain parity. We notice how, when relaxation is slow (light green curve), the two-state model overestimates fidelity by one order of magnitude due to misclassified |T_0_〉. In **c**, we show with dashed lines the points of the highest (yellow curve, *F*_m_ = 99.92% in 340 μs) and fastest (red curve, *F*_m_ = 99% in 24 μs) fidelity. **d**, Ratio of relaxation times for triplet states |T_−_〉 and |T_0_〉. This shows the optimal point for parity readout is at the maximum ratio of relaxation times for *V*_GTB2_ = 213 mV (yellow arrow), whereas fast readout is achievable for overall shorter relaxations (red arrow). Data were acquired from 10,000 shots. For rates, error bars represent ±1*σ* from the fit to an exponential decay. For fidelities, error bands approximate to ±1*σ* from the fit in **b**, and range between minima and maxima from the fits in **c** and **d**, as detailed in Methods.

After SNR improvement, the two-state model of equation (1) predicts that spin readout can be maximized by optimizing the relevant triplet relaxation times. This can be achieved by adjusting the DQD barrier gate voltage, which controls tunnel coupling between the two dots, as shown in the inset of Fig. 4a, although only over a narrow range due to thermal broadening (Supplementary Note I). We directly measure the relaxation rates of both |T_0_〉 and |T_−_〉 in Fig. 4a and observe an exponential dependence on barrier voltage. In particular, we see how reducing the barrier voltage and thus *t*_c_ strongly suppresses triplet relaxation as the dots decouple^40^. This tunability, spanning almost three orders of magnitude in relaxation rate, enables a study of readout fidelity.

We first calculate the parity readout fidelity ${F}_{{\rm{m}}}^{\ast }$ using the two-state model, as shown in Fig. 4b. Fidelity initially increases with *t*_read_ due to an improved SNR, until it decreases as triplet relaxation introduces errors. It is maximized at the barrier voltage corresponding to slow relaxation due to the ability to increase *t*_read_ and suppress noise, as encapsulated in equation (1). However, the two-state model becomes inaccurate when ${t}_{{\rm{read}}}\sim {\varGamma }_{{{\rm{T}}}_{0}}^{-1}$, which occurs between 2 and 200 μs over the range of barrier gates studied here. In this regime, |T_0_〉 states, which have odd parity, have not fully decayed, and the presence of this third spin state leads to a significant misclassification. The result is an overestimation of the readout fidelity, thus requiring a more comprehensive model that includes |T_0_〉 (see Fig. 7 in Methods).

We therefore use a HMM with three states as introduced above to model the distribution (still using the threshold method for classification). This yields a more accurate measure of fidelity *F*_m_, as shown in Fig. 4c. First, the maximum fidelity is lower than ${F}_{{\rm{m}}}^{\ast }$ as the two-state model did not account for undecayed |T_0_〉 states. This is particularly visible at slow relaxation rates (low barrier voltage), where the slow |T_0_〉 decay increases the risk of misclassification as |T_−_〉, and so increases parity readout errors. Second, including the full distribution changes the optimal tuning point. Although fidelity usually increases as relaxation rates decrease, optimal parity readout actually occurs when the distinguishability |T_0_〉 and |T_−_〉 is maximized. This corresponds to a maximal ratio ${\varGamma }_{{{\rm{T}}}_{0}}/{\varGamma }_{{{\rm{T}}}_{-}}$ (Fig. 4d), where we obtain a parity readout fidelity of *F*_m_ = 99.92% in 340 μs.

**Extended Data Fig. 1:**
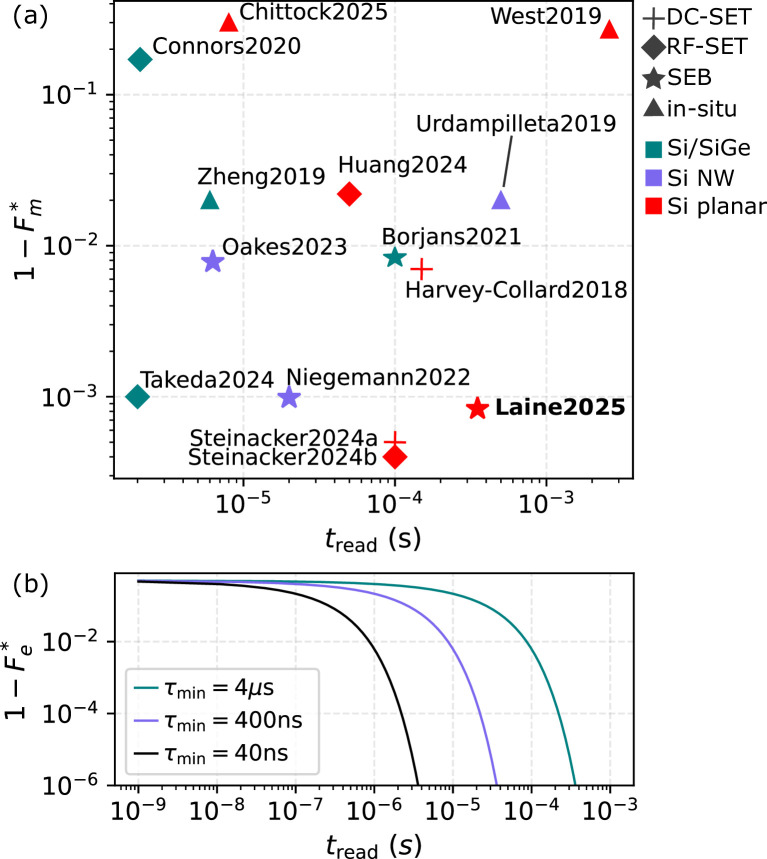
Spin readout fidelity in the literature and possible improvement. (**a**) Spin readout fidelity using Pauli Spin Blockade across platforms and sensors. The data and references can be found in Extended Table 1. (**b**) Electrical fidelity plotted from Eq. (12) with voltage SNR taken as ${\rm{S}}NR=\sqrt{{t}_{{\rm{read}}}/{\tau }_{\min }}$. The spin ${\tau }_{\min }$ for this work is estimated to ${\eta }^{-1}\times {\tau }_{\min } \sim 4.1\mu$ s (Appendix I) which incorporates the loss of visibility *η*^−1^ due to distinguishing (0, 2) and (1, 1) Coulomb peaks when spin sensing. This shows that reducing our current SEB ${\tau }_{\min }$ by an order of magnitude would allow to reach readout fidelity of 99% under 10*μ*s.

Some quantum computing applications may prioritize measurement speed over fidelity, provided the latter exceeds some threshold, and our results show how the relaxation rates can be adjusted to optimize for specific targets. Because we require ${\varGamma }_{{{\rm{T}}}_{0}}^{-1} < {t}_{\mathrm{read}} < {\varGamma }_{{{\rm{T}}}_{-}}^{-1}$, increasing the measurement speed while maintaining high fidelity means increasing relaxation rates. For example, a fidelity of *F*_m_ = 99% in 24 μs can be achieved by increasing the barrier gate voltage, as shown in Fig. 4c. Even in noisy intermediate-scale quantum (NISQ)-type applications where readout occurs only at the end of a circuit, speed and fidelity must be balanced to maximize the repetition rate without substantially increasing the overall error rate. For fault-tolerant architectures requiring mid-circuit readout, a short readout time becomes critical and must be much less than both the qubit relaxation time *T*_1_ and the Hahn echo decoherence time *T*_2_ (~28 ms for Si-MOS QDs^22^) or else ${T}_{2}^{\ast }$ (~20−100 μs). The speed demonstrated here is already compatible with such requirements (assuming refocusing pulses are used), and further improvements in charge-readout SNR (see Extended Data Fig. 1) could reduce the measurement time further.

### Spin classification beyond the threshold method

**Fig. 5:**
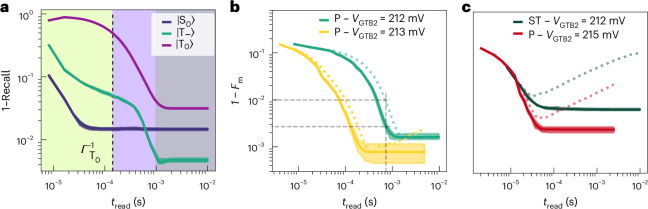
Classification using the HMM. **a**, Recall (proxy to readout fidelity) for each state as a function of the readout time, using the HMM to model the data and to classify traces at *V*_GTB2_ = 212 mV. When ${t}_{{\rm{read}}}\ll {\varGamma }_{{{\rm{T}}}_{0}}^{-1}$ (light green region), the recall of |T_−_〉 and |S〉 improves as the readout time increases because noise averages out, while unrelaxed |T_0_〉 is misidentified as |T_−_〉. Once ${t}_{{\rm{read}}}\ge {\varGamma }_{{{\rm{T}}}_{0}}^{-1}$ (purple region), |T_0_〉 becomes distinguishable from |T_−_〉, improving recall for both triplets. Finally, for ${t}_{{\rm{read}}}\gg {\varGamma }_{{{\rm{T}}}_{-}}^{-1}$ (grey region), all triplet states have decayed and the recall plateaus because no extra information remains. **b**,**c**, Readout fidelity from the HMM-based classifier (solid lines) compared with the threshold classifier (dashed lines). **b**, Parity readout (labelled P). The HMM outperforms the threshold method when ${t}_{{\rm{read}}} < {\varGamma }_{{{\rm{T}}}_{0}}^{-1}$ because it tracks the T_0_-to-S decay, while the threshold method keeps confusing T_0_ with T_−_. At the optimal point (*V*_GTB2_ = 212 mV), the fidelity increases from 99% to 99.5%, as indicated by the grey dashed lines. **c**, The HMM fidelity stays stable once *t*_read_ exceeds the tunnelling rates, while the threshold method starts to fail because the averaged signal picks up a growing singlet segment. This is evident in the singlet–triplet readout (labelled ST) at *V*_GTB2_ = 212 mV when ${t}_{\mathrm{read}} > {\varGamma}_{{{\rm{T}}}_{0}}^{-1}=170\,{\mu{\mathrm{s}}}$ (dark green plot). The parity readout at *V*_GTB2_ = 215 mV (red plot) behaves the same way. In this case |T_0_〉 is irrelevant, because it decays before the smallest readout time, so the dynamics are governed by ${\varGamma }_{{{\rm{T}}}_{-}}^{-1}\simeq 200\,{\mu {\mathrm{s}}}$. As the integration time increases, the threshold fidelity keeps dropping while the HMM remains stable. For fidelities, error bands range between minima and maxima resulting from fit error bars, as calculated in Methods.

Finally, we explore whether spin readout fidelity and speed can be improved beyond the threshold method by using the full information contained in a single-shot trace^26,32^. We use the forward–backwards algorithm based on the HMM to classify a single-shot trace without averaging (Methods). Exploiting the different relaxation rates of the |T_0_〉 and |T_−_〉 states, we discriminate all three prepared states |S〉, |T_0_〉 or |T_−_〉. Accurate classification requires waiting for all the states to decay but one, as this produces a characteristic signature of the time trace. This effect of readout time on readout accuracy is visible when measuring the recall, defined as the ratio of correctly identified traces for a given state to the total number of occurrences of that state, which serves as the best proxy for the single-state measurement fidelity *F*_m_ (Methods). In Fig. 5a, we calculate the recall of each state in the regime of longest triplet relaxation rates, where the effect of undecayed |T_0_〉 states is most pronounced. At short readout times ${t}_{\mathrm{read}}\ll {\varGamma }_{{{\rm{T}}}_{0}}^{-1}$ it is impossible to distinguish |T_0_〉 from |T_−_〉. Classification becomes possible only once the odd-parity triplets begin to decay (${t}_{{\rm{read}}} > {\varGamma }_{{{\rm{T}}}_{0}}^{-1}$), which further benefits the readout of |T_−_〉. In contrast to the threshold method, fidelity does not decrease at long readout times because the HMM effectively acts as a nonlinear Bayesian filter^26^. Such an analysis highlights the key difference between classifying single-shot traces with the threshold method and the forward–backwards algorithm, and indicates the optimal measurement time depending on which state is most relevant for computation.

After classifying single-shot traces into three states, the results can be combined to produce binary readout in either the singlet–triplet or parity basis. In some regimes, this offers an improvement in fidelity and speed over the threshold method, as shown in Fig. 5b,c. Speed improvement occurs for slow |T_0_〉 relaxation, where the binary thresholding struggles to distinguish between three states. In this case, the forward–backwards algorithm enables up to a twofold reduction in error rate for the same readout time (Fig. 5b). In the case of fast relaxation, the algorithm instead improves overall fidelity because it is no longer limited by decay at long readout times (Fig. 5c).

## Discussion

This demonstration of an SEB in a planar MOS architecture establishes that a high-fidelity, scalable charge sensor can be realized on an industrial-grade platform. This compatibility with foundry processing offers potential advantages in yield and reproducibility (suggested by preliminary results on tuning of multiple SEBs in devices produced by the same process), while retaining the low charge noise ($10\,{\mu {\mathrm{eV}}}\,{\mathrm{Hz}}^{-1/2}$ at 0.5 mHz; Supplementary Note H) observed in state-of-the-art academic devices^41^.

**Fig. 6:**
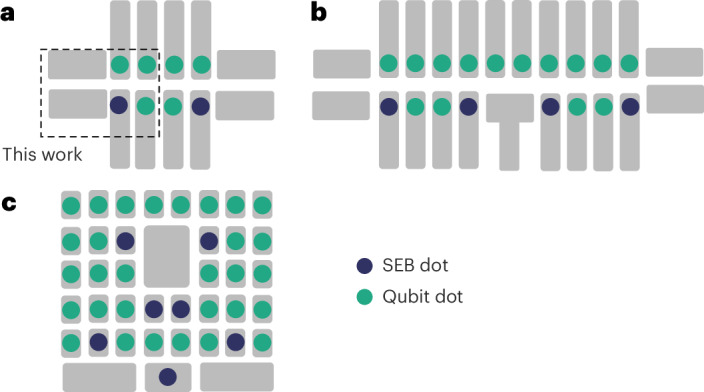
QPU architectures containing the SEB. **a**, A 2 × 4 array of QD architecture based on the unit cell presented in this work (dashed square). Notice that in the device studied in this work, the qubit dot positioned in the same row as the sensor dot was non-functional. **b**, A 2 × *N* array with T-shape reservoir where sensor and qubits can be placed on the lower row. The optimal number of qubit dots between sensors in the bottom row remains to be determined and will depend on the quality of the sensor. **c**, An *N* × *N* array with island reservoirs leading to higher qubit connectivity. The size of the reservoir gate would also have to be optimized: a small reservoir could lead to the formation of elongated dots^57^, whereas increasing the reservoir size means losing connectivity.

The compact nature of the SEB also allows it to be integrated into tileable unit cells of scaled QPU architectures, as illustrated in Fig. 6. A key advantage over previous MOS demonstrations is that the sensors are no longer confined to the edge of the qubit array, which often limits sensitivity when scaling to longer chains of dots. A similar geometry could be achieved by placing SETs in a parallel channel, as demonstrated in SiGe (ref. ^42^), but the requirement for two SET reservoirs prevents qubits from being placed in the same channel as the sensors, because impedance changes co-linear with the sensor axis are screened by the large reservoirs. Instead, SEBs allow qubits to be positioned in both channels, increasing qubit connectivity, as shown in Fig. 6a,b. Beyond 2 × *N* structures, SEB sensors could also be deployed within an *N* × *N* dot array, as illustrated in Figs. 6c. In such architectures, including those fabricated using the single-layer etched-defined electrodes (SLEDGE) process^43^, forming island reservoirs could be challenging but possibly achieved using localized doping or accumulation gates with charges implanted via initial illumination.

**Fig. 7:**
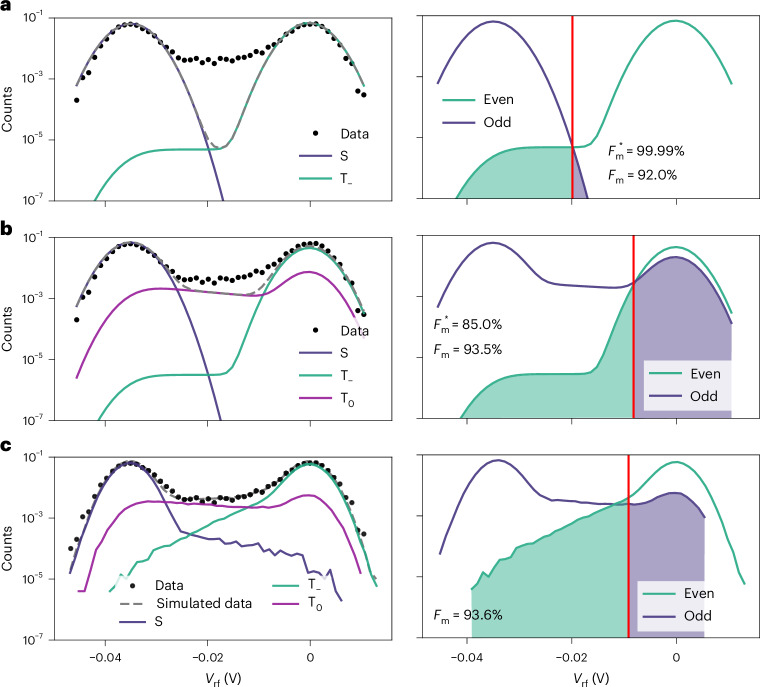
Comparison of data simulation for fidelity calculation. **a**–**c**, Left column: histograms of 10,000 single-shot data (points) integrated for *t*_read_ = 204 μs, with ${\varGamma }_{{{\rm{T}}}_{0}}^{-1}=170\,{\mu {\mathrm{s}}}$ and ${\varGamma }_{{{\rm{T}}}_{-}}^{-1}=230\,{\rm{ms}}$, as well as the simulated distribution (line) using three different methods. Histograms are normalized, and the *x* axis is the raw RF signal. Right column: same histograms with distributions binned into odd (|S〉, |T_0_〉) and even (|T_−_〉) states. The optimal threshold voltage for parity readout (red line) is found by minimizing the readout error (shown as the shaded region) for each simulation method. The threshold is used to calculate fidelities. ${F}_{{\rm{m}}}^{\ast }$ is calculated from the analytical distribution (using equation (10) for **a**, equation (15) for **b**) and *F*_m_ from the ‘real’ distribution found in **c** (equation (19)). **a**, The data are fitted to a two-state model via equation (6). The threshold is optimized for ${F}_{{\rm{m}}}^{\ast }$. We find that ${F}_{{\rm{m}}}^{\ast } > {F}_{{\rm{m}}}$ and thus the two-state model greatly overestimates the fidelity, neglecting the undecayed odd-parity triplets. **b**, We extend to a three-state model, which accounts for |T_0_〉 states, improving the fit but still not capturing all the data. The optimal threshold voltage is shifted up to minimize the error due to |T_0_〉 classified as even parity. We find that (1) ${F}_{{\rm{m}}}^{\ast } < {F}_{{\rm{m}}}$, showing that this model underestimates fidelity as it mistakes TLF and accounts for more |T_0_〉 states than there is, and (2) *F*_m_ is larger than in **a** because it corresponds to a more optimal threshold. **c**, The data are simulated using an HMM that contains |T_0_〉 and the TLF, and captures the data well. We note that classifying the single-shot traces with the HMM and not with the threshold method gives an improved fidelity of *F*_m_ = 95.9% for that same integration time.

This work has also explored the use of the HMM for more accurate state simulation and classification. Such a method is particularly effective when readout involves more than two states that produce the same signal but differ in duration (for example, from different decay rates). In this case, traditional thresholding is limited to a binary output and cannot distinguish states unless they decay long enough to affect the averaged signal, reducing the measurement speed. Our algorithm could be extended to classifying the |T_+_〉 state, provided its relaxation rate differs from the other triplets, which could offer more efficient state initialization by reducing the number of readout attempts^44,45^. Moreover, this method may enable on-the-fly measurements with adaptive integration times^46^, where integration stops once the predicted qubit state reaches a confidence threshold, thus reducing the readout time. This capability is particularly relevant for quantum error correction, where the optimal trade-off between speed and fidelity can vary depending on the dominant noise in a stabilizer circuit (for example, idling noise of data qubits versus measurement errors^47^). Finally, going beyond spin qubits, a readout protocol capable of discriminating between multiple states could be particularly useful when measurable states extend beyond the computational subspace, such as leakage states^48–50^. In summary, this work has shown that the combination of an SEB and HMM-based readout can be used to realize a compact charge sensor operating at speeds of 10–300 kHz. This is an important step towards building a scalable QPU 2D architecture, and has potential applications for capacitive sensing beyond spin qubits.

## Methods

### Fidelity calculation using an analytical model

#### Two-state model

The histogram obtained from repeated single-shot measurements corresponds to two peaks associated with each charge state (1, 1) and (0, 2) separated by the signal strength δ*V*_rf_ and broadened equally by Gaussian noise, which holds when the noise is dominated by the noise temperature of the high electron mobility transistor (HEMT) amplifier^51^. In the simplest case, we can model the data by assuming the two charge states correspond to two distinct spin states. This causes an asymmetry in the distribution due to relaxation from the excited (triplet) (1, 1) state into the singlet (0, 2) state, further broadening the triplet peak. Fidelity is calculated by fitting the experimental histogram to this bimodal distribution and finding an optimal threshold voltage that minimizes the classification error, that is, the overlap of the underlying distributions of the two states being discriminated.

Specifically, the singlet state generates white noise (*σ*(*t*)) centred at the singlet voltage *V*_S_. This results in a signal distribution *n*_S_(*V*, *t*) at integration time *t* = *t*_read_, described by the following equation:

3 $${n}_{{\rm{S}}}(V,\,t)=\frac{1}{\sqrt{2\pi }\sigma (t)}\exp \left(-\frac{{(V-{V}_{{\rm{S}}})}^{2}}{2\sigma {(t)}^{2}}\right)$$

In the presence of Gaussian noise, the standard deviation decreases as a function of the integration time *t*. We can write the standard deviation as $\sigma (t)={\sigma }_{0}\sqrt{\frac{{t}_{0}}{t}}$, where *σ*_0_ is the standard deviation at the reference integration time *t*_read_ = *t*_0_.

For a triplet state, the signal remains at the triplet voltage *V*_T_ for a time *T*_1_ = *Γ*^−1^ before decaying to the singlet state. Here we do not make any assumption on the triplet state at stake, and consider that only one triplet state is to be considered, for example, the |T_−_〉 state for parity readout (considering all |T_0_〉 has relaxed) or the |T_0_〉 state for singlet–triplet readout. The triplet distribution is modelled as

4 $${n}_{{\rm{T}}}(V,\,t)=\frac{1}{\sqrt{2\pi }\sigma (t)}\exp \left(-\frac{t}{{T}_{1}}\right)\exp \left(-\frac{{(V-{V}_{{\rm{T}}})}^{2}}{2\sigma {(t)}^{2}}\right)+{I}_{{\rm{D}}}$$

where *I*_D_ represents the tail that forms due to short-lived triplets decaying into a singlet. Such a tail is given by^26,37,52^

5 $$\begin{array}{l}\begin{array}{l}\begin{array}{l}{I}_{{\rm{D}}}=\frac{t}{{T}_{1}}\frac{1}{\sqrt{8\pi }({V}_{{\rm{T}}}-{V}_{{\rm{S}}})}\exp \left[\frac{t}{{T}_{1}}\frac{1}{{V}_{{\rm{T}}}-{V}_{{\rm{S}}}}\left({V}_{{\rm{S}}}-V+\frac{{\sigma }^{2}(t)}{2({V}_{{\rm{S}}}-{V}_{{\rm{T}}})}\frac{t}{{T}_{1}}\frac{1}{{V}_{{\rm{T}}}-{V}_{{\rm{S}}}}\right)\right]\\ \left[\mathrm{erf}\left(\frac{t}{{T}_{1}}\frac{\sigma (t)}{\sqrt{2}({V}_{{\rm{S}}}-{V}_{{\rm{T}}})}+\frac{V-{V}_{{\rm{S}}}}{\sqrt{2}\sigma (t)}\right)-\mathrm{erf}\left(\frac{t}{{T}_{1}}\frac{\sigma (t)}{\sqrt{2}({V}_{{\rm{S}}}-{V}_{{\rm{T}}})}+\frac{V-{V}_{{\rm{T}}}}{\sqrt{2}\sigma (t)}\right)\right]\end{array}\end{array}\end{array}$$

Equations (3) and (4) allow us to identify the parts of the histogram corresponding to each spin state. We can then fit the raw data histogram to the combined distribution:

6 $$n(V,\,t)={P}_{{\rm{T}}}\times {n}_{{\rm{T}}}(V,\,t)+{P}_{{\rm{S}}}\times {n}_{{\rm{S}}}(V,\,t)$$

where *P*_S_ and *P*_T_ are the fractions of traces in the singlet and triplet states, respectively, with *P*_S_ + *P*_T_ = 1. Figure 7a shows the fit of equation (6) to the histogram of single-shot data.

Using the analytical formula, fidelities for the singlet state ${F}_{{\rm{S}}}^{\ast }({V}_{{\rm{th}}})$ and the triplet state ${F}_{{\rm{T}}}^{\ast }({V}_{\mathrm{th}})$ can be calculated as the fraction of events misclassified given the threshold voltage *V*_th_:

7 $${F}_{{\rm{S}}}^{\ast }({V}_{\mathrm{th}})=1-{\int }_{{V}_{\mathrm{th}}}^{\infty }{n}_{{\rm{S}}}(V)\,{\rm{d}}V\,\mathrm{and}$$

8 $${F}_{{\rm{T}}}^{\ast }({V}_{\mathrm{th}})=1-{\int }_{-\infty }^{{V}_{\mathrm{th}}}{n}_{{\rm{T}}}(V)\,{\rm{d}}V$$

The fidelity metric assumes equal probabilities for singlet and triplet states (*P*_T_ = *P*_S_ = 0.5), treating both types of misclassification equally. Among these metrics, visibility ${V}_{{\rm{m}}}^{\ast }$ is defined as

9 $${V}_{{\rm{m}}}^{\ast }= \max _{{V}_{\mathrm{th}}}({F}_{{\rm{S}}}^{\ast }({V}_{\mathrm{th}})+{F}_{{\rm{T}}}^{\ast }({V}_{\mathrm{th}})-1)$$

which can take values between zero and one.

Alternatively, the measured fidelity ${F}_{{\rm{m}}}^{\ast }$ determines the average error in assigning the wrong label to the measured state and thus takes values between 0.5 and 1:

10 $${F}_{{\rm{m}}}^{\ast }=\mathop{{\max }}\limits_{{V}_{\mathrm{th}}}\left(\frac{{F}_{{\rm{S}}}^{\ast }({V}_{\mathrm{th}})+{F}_{{\rm{T}}}^{\ast }({V}_{\mathrm{th}})}{2}\right)$$

This is the metric usually quoted in the literature as *F*_m_. The maximum measured fidelity will occur when *n*_S_(*V*_th_) = *n*_T_(*V*_th_), which means that we can also define ${F}_{{\rm{m}}}^{\ast }$ as

11 $${F}_{{\rm{m}}}^{\ast }=\frac{1}{2}\left[1-\mathrm{erf}\left(\frac{{V}_{{\rm{S}}}-{V}_{\mathrm{th}}}{\sqrt{2}\,\sigma }\right)\right]$$

In Fig. 7, we visualize the parity readout error by the shaded area, which corresponds to the integral of the even (respectively odd) density below (above) threshold, coloured in green (purple). In Fig. 7a we calculate a spin parity measurement fidelity of 99.99%; however, this value is probably overestimated, as indicated by the mismatch between the data and the fitted histograms. The intersection of the peaks in the model is smaller than observed in the experimental data, which can be attributed to the presence of |T_0_〉 spin states and a two-level fluctuator, factors not accounted for and leading to an inflated fidelity estimate.

#### Electrical fidelity

We also note the definition of the electrical fidelity, which corresponds to the trivial case for which the relaxation time is taken as infinite and thus *V*_th_ will be the average between *V*_S_ and *V*_T_. This gives an expression for the electrical fidelity *F*_e_ that does not account for spin–relaxation errors:

12 $$\begin{array}{l}\begin{array}{l}\begin{array}{l}{F}_{{\rm{e}}}^{\ast }=\mathop{\,\mathrm{lim}}\limits_{{{\rm{T}}}_{1}\to \infty }{F}_{{\rm{m}}}^{\ast }=\frac{1}{2}\left[1+\mathrm{erf}\left(\frac{{V}_{{\rm{T}}}-{V}_{{\rm{S}}}}{2\sqrt{2}\,\sigma }\right)\right]\\ =\frac{1}{2}\left[1+\mathrm{erf}\left(\frac{\mathrm{SNR}}{2\sqrt{2}}\right)\right]\end{array}\end{array}\end{array}$$

The electrical fidelity is useful for discerning the nature of the readout errors. $1-{F}_{{\rm{e}}}^{\ast }$ gives the errors due to the sensor, and ${F}_{{\rm{e}}}^{\ast }-{F}_{{\rm{m}}}^{\ast }$ are the relaxation errors.

#### Three-state model

To refine the fit of equation (6) and account for undecayed |T_0_〉 spin states, we can add a third distribution ${n}_{{{\rm{T}}}_{0}}$ with probability ${P}_{{{\rm{T}}}_{0}}$ where ${P}_{{\rm{S}}}+{P}_{{{\rm{T}}}_{-}}+{P}_{{{\rm{T}}}_{0}}=1$. For parity readout, we now define

13 $${F}_{\mathrm{odd}}^{\ast }({V}_{\mathrm{th}})=1-{\int }_{-\infty }^{{V}_{\mathrm{th}}}({n}_{{\rm{S}}}+{n}_{{{\rm{T}}}_{0}})\,{\rm{d}}V\,\mathrm{and}$$

14 $${F}_{\mathrm{even}}^{\ast }({V}_{\mathrm{th}})=1-{\int }_{{V}_{\mathrm{th}}}^{+\infty }{n}_{{{\rm{T}}}_{-}}\,{\rm{d}}V$$

and therefore measurement fidelity is calculated as

15 $${F}_{{\rm{m}}}^{\ast }=\mathop{{\max }}\limits_{{V}_{\mathrm{th}}}\left(\frac{{F}_{\mathrm{odd}}^{\ast }({V}_{\mathrm{th}})+{F}_{\mathrm{even}}^{\ast }({V}_{\mathrm{th}})}{2}\right)$$

To avoid biasing classification, we fix ${P}_{{\rm{S}}}=0.25,\,{P}_{{{\rm{T}}}_{0}}=0.25,\,{P}_{{{\rm{T}}}_{-}}=0.5$ in the case of parity readout.

We calculate the density ${n}_{{{\rm{T}}}_{0}}$ similarly to that of equation (4), changing to the appropriate relaxation time. When fitting the data to this overall distribution, we notice a subsequent improvement to the fit quality as seen in Fig. 7b. This is expected, as the histogram is measured at *t*_read_ = 204 μs, a value comparable to ${\varGamma }_{{{\rm{T}}}_{0}}^{-1}=168\,{\mu {\mathrm{s}}}$, therefore leaving a substantial number of undecayed *T*_0_ states. However, the three-state model is still insufficient to fully capture the data, especially between the two peaks. As we explain in the next section, this is due to the effect of a two-level fluctuator visible in the shoulder of the singlet distribution towards increasing *V*_rf_.

### Fidelity calculation with simulated data using an HMM

To measure the classification fidelity of the HMM, we first simulate the data and fine-tune the HMM’s free parameters, as described in Supplementary Note F. The fidelity for each spin state is calculated as

16 $${F}_{{\rm{S}}}=1-\frac{{\rm{Singlets}}\,{\rm{misclassified}}}{{\rm{Total}}\,{\rm{number}}\,{\rm{of}}\,{\rm{predictions}}}\,{\rm{and}}$$

17 $${F}_{{\rm{T}}}=1-\frac{{\rm{Triplets}}\,{\rm{misclassified}}}{{\rm{Total}}\,{\rm{number}}\,{\rm{of}}\,{\rm{predictions}}}$$

The overall fidelity is then calculated as the average fidelity across all spin states:

18 $${F}_{{\rm{m}}}=\frac{1}{N}\displaystyle \mathop{\sum }\limits_{i=1}^{N}{F}_{i}$$

For two spin states, this simplifies to

19 $${F}_{{\rm{m}}}=1-\frac{{\rm{Number}}\,{\rm{of}}\,{\rm{errors}}}{2\times ({\rm{Total}}\,{\rm{number}}\,{\rm{of}}\,{\rm{predictions}})}$$

Similarly to before, such fidelity is calculated at each threshold voltage, and the overlap of the states being discriminated (for example, odd/even parity) is minimized by setting the optimum threshold value.

Here we note that although fidelity is widely used, it has substantial limitations. For example, when all classifications are systematically incorrect, the minimum value of *F*_m_ depends on the number of states. With two spin states, the minimum fidelity is *F*_m_ = 0.5, whereas for three states, it is *F*_m_ = 0.66. This inconsistency makes fidelity a poor metric for systems with more than two states.

Visibility addresses these shortcomings. Defined analogously to accuracy in machine learning, visibility measures the proportion of correctly classified events:

20 $${V}_{{\rm{m}}}=1-\frac{{\rm{Number}}\,{\rm{of}}\,{\rm{errors}}}{{\rm{Total}}\,{\rm{number}}\,{\rm{of}}\,{\rm{predictions}}}$$

Unlike fidelity, visibility is consistent across systems with any number of spin states and can naturally extend to applications involving qudits or other multi-state systems^53,54^. This makes it a more reliable and versatile metric for evaluating classification performance. However, we have used fidelity as our metric for consistency and comparison with the literature.

### Classification methods

In this Article we use two methods to classify readout traces: the widely used threshold technique and a more advanced approach based on an HMM.

#### Threshold

The threshold method is a simple and widely used technique for labelling spin states. The signal is averaged over a set integration time (*t*_read_), and the spin state is determined by comparing this average to a predefined threshold^37^. This is the threshold we refer to when calculating readout fidelity.

#### Hidden Markov model

In the main text and when calculating fidelity, we describe how an HMM can represent the dynamics of our system. The HMM can model the hidden states (for example, spin configurations) and relate them to the observed sensor signal.

The forward–backwards algorithm calculates the likelihood that the system is in a specific hidden state at any time *t*, based on the observed signal up to *t* and also data measured after *t*, up to the readout time. This probability is given by

21 $$p({z}_{t}|{y}_{1:{\rm{T}}},\,\theta ),$$

where *z*_*t*_ is the hidden state at time *t*, *y*_1:T_ is the observed signal over the entire measurement, and *θ* is the HMM parameters, including initial state probabilities, the transition matrix and emission probabilities.

For classifying spin states, we use the forward–backwards algorithm to determine the most likely hidden state at the start of the measurement (*t* = 0). This is achieved by maximizing the probability:

22 $$\mathrm{predicted}\,\mathrm{state}=\arg \max _{{z}_{0}}\,p({z}_{0}|{y}_{1:T},\,\theta )$$

The HMM can also operate in real time by continuously updating the probability of the initial state, *p*(*z*_0_∣*y*_1:*T*_, *θ*), as data are acquired. The measurement can stop once this probability exceeds a chosen confidence threshold, for example, 99.5%. This adaptive approach can be implemented on low-latency hardware such as a field-programmable gate array.

Unlike the threshold method, this approach takes advantage of the entire time-dependent structure of the observed data collected during the integration period (*t* = *t*_read_), providing improved classification accuracy.

We implement this analysis using the Python library dynamax^55^, which offers efficient tools for HMM parameter estimation and state inference^56^. The data simulation for our system that incorporates the three spin states and a two-level fluctuator is detailed in Supplementary Note F.

### Fidelity calculations across datasets

In this study we have used different methods to calculate measurement fidelity depending on how the data are simulated and how it is classified, which we summarize in Table 1.

**Table 1:** Summary of methods used for fidelity calculations

Dataset	Label	Simulation model	Classification method	Fidelity equation
Fig. 3c	${F}_{{\rm{e}}}^{\ast }$	Two-state	Threshold	Equation (12)
Fig. 4b	${F}_{{\rm{m}}}^{\ast }$	Two-state	Threshold	Equation (15)
Fig. 4c	*F*_m_	HMM	Threshold	Equation (19)
Fig. 5	*F*_m_	HMM	HMM	Equation (19)

In Fig. 4b, the uncertainty in the fidelity is estimated by evaluating the fidelity function at the fit parameters values offset by ±1 standard deviation—the fit corresponding to the two-state model. This provides an approximate 1*σ* error bar on the fidelity, assuming it depends linearly on the parameters for small variations.

In Fig. 4c, when data are simulated with the HMM and classified with the threshold method, we generate five datasets of 60,000 readout traces at each barrier gate voltage. The first 10,000 traces are used to choose a threshold voltage. For each integration time (*t*_read_) we take the mean of the trace during *t*_read_ and select the best threshold. The rest of the 50,000 traces are used to calculate fidelity, as detailed in Supplementary Note F. The error bars shown in the figure represent the maximum and minimum fidelity across the five datasets, in a qualitative estimate of uncertainty.

In Fig. 5, when data are simulated with the HMM and classified with the HMM, we generate five datasets of 50,000 readout traces at each barrier gate voltage. There is no need to retrain the HMM, so the traces are used to calculate fidelity. The error bars shown in the figure are also given by the maximum and minimum fidelity across the five datasets.

### Reporting Summary

Further information on research design is available in the Nature Portfolio Reporting Summary linked to this Article.

## Supplementary information

Supplementary Information Supplementary discussion

Reporting Summary

## Figures and Tables

**Extended Data Fig. 2:**
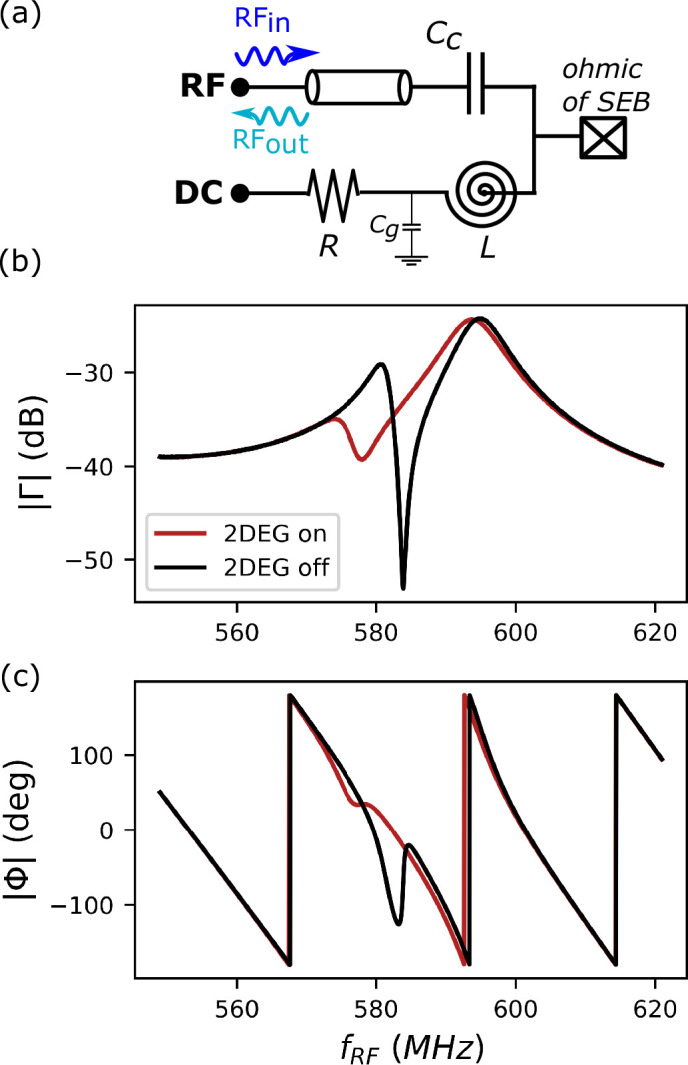
Readout resonator and its characterisation. (**a**) Parallel LC circuit used for reflectometry. (**b,c**) VNA traces taken at 10mK, when bottom reservoir gate is at to turn-on voltage point and electron 2DEG is accumulated (red line) and when all gate voltages are at zero (black line).

**Extended Data Fig. 3:**
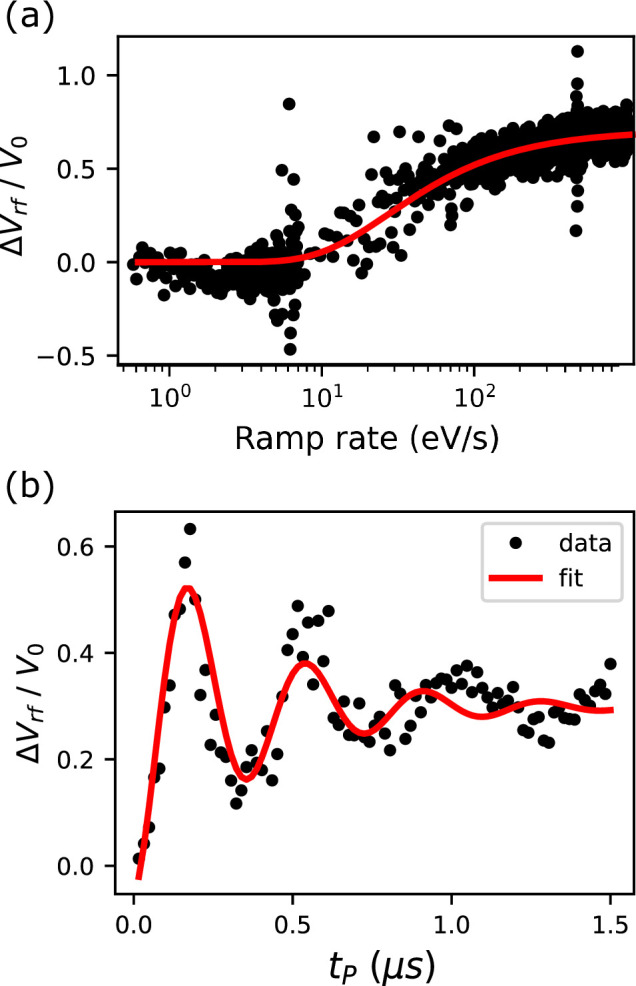
Characterisation of the qubit dots. (**a**) LZ single passage experiment, where ramp rate is varied across the ${\Delta }_{S-{T}_{-}}$ anticrossing. Fit to Eq. 7 with ${\Delta }_{S-{T}_{-}}=46.9\pm 0.9$ neV. (**b**) Measurement of dephasing time from coherent oscillation measured in the singlet–triplet basis. The oscillation is fitted to a damp sine wave with *T*_2_ = 0.4 ± 0.1*μ*s, *f*_*Rabi*_ = 17.0 ± 0.4 MHz and *A* = 0.35 ± 0.04 fitted to Eq. 8.

**Extended Data Fig. 4:**
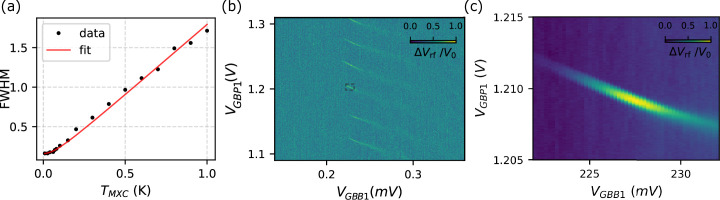
Characterisation of the SEB dot. (**a**) SEB thermometry. Measurement of the SEB FWHM as a function of temperature and fit to Eq. 9 to extract *T*_*e*_ = 90 ± 11 mK and *α* = 0.17 ± 0.01. (**b**) Stability map showing six DRT where the lowest electron number (*V*_GBP1_ < 1.1V) is 15. The dashed rectangle corresponds to the range used for SNR optimisation in Fig. 3b of the main text. (**c**) Zoom on the operating region where the SNR is optimised as in Fig. 3b of the main text. Notice that the stability map of Fig. 3a of the main text corresponds to a different thermal cycle and is obtained at lower power, which explains some voltage shifts.

**Extended Data Fig. 5:**
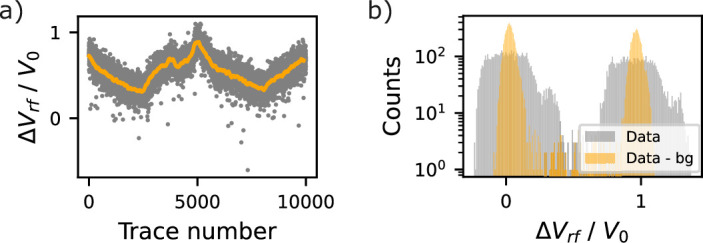
Data Pre-Processing for Readout. (**a**) Sensor drift observed during the readout fidelity process (shown in grey), whose fluctuations are mitigated by applying a moving average filter with a window size of 50, as shown in orange. The interval between consecutive readout traces depends on the duration of the entire readout sequence, which includes an initialization phase requiring a wait time of approximately five times *T*_1_. This example corresponds to the longest *T*_1_, resulting in the longest readout sequence, lasting nearly one second. (**b**) Histograms comparing 10,000 single-shot traces averaged over *t*_int_ = 204 μs before (grey) and after (orange) background effect removal.

**Extended Data Fig. 6:**
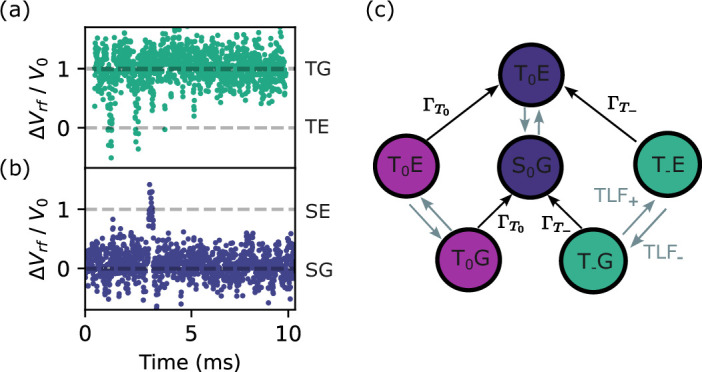
TLF effect on Sensor Signal. (**a**) Effect of the two-level fluctuator (TLF) on the sensor signal for the singlet state $(\; | {\rm{S}}\rangle$). When the TLF is in the ground state (G), the sensor signal is centred at *ΔV*_rf_/*V*_0_ = 0. However, when the TLF switches to the excited state (E), the signal shifts to resemble a triplet state (*ΔV*_rf_/*V*_0_ = 1). (**b**) Effect of the two-level fluctuator (TLF) on the sensor signal for the triplet states $(\; | {{\rm{T}}}_{-}\rangle$). (**c**) Markov chain representing the six hidden states, including the transitions of the two-level fluctuator: TLF_+_ for transitions from the ground (G) to the excited (E) state, and TLF_−_ for transitions from the excited (E) to the ground (G) state.

**Extended Data Fig. 7:**
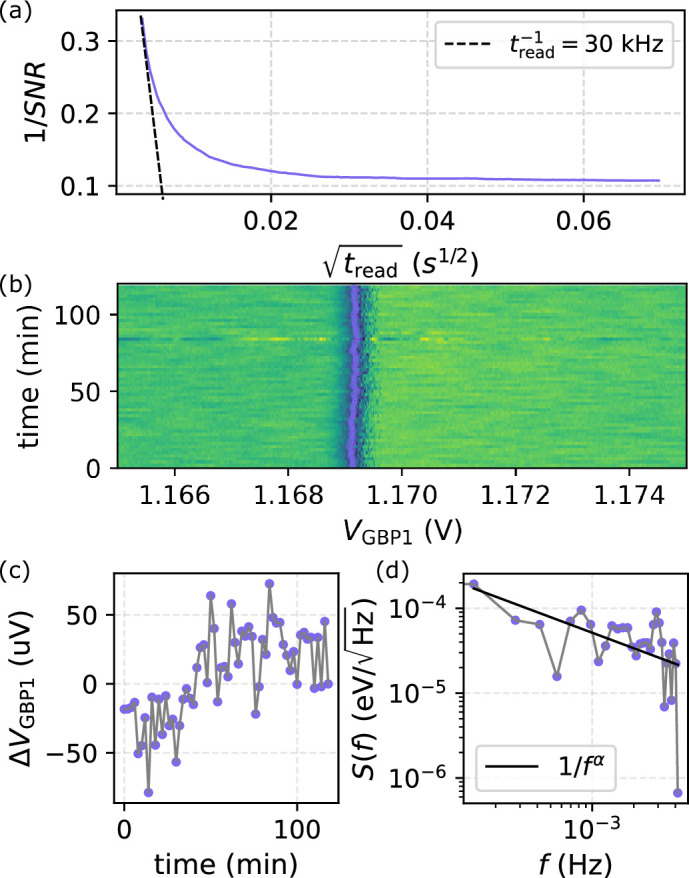
Noise analysis. (**a**) Fast noise: at fixed signal 1/SNR = *σ* is suppressed linearly with $1/\sqrt{{t}_{{\rm{read}}}}$ up until it plateau for ${t}_{{\rm{read}}}^{-1} \sim 30$ kHz. This is where non-white noise such as charge noise starts to dominate. (**b**) Stability of the SEB: peak tracking of the SEB point with centre obtained from fit to c*osh*^−2^. (**c**) Slow noise : noise spectral density calculated from welch method and using gate lever-arm *α*_*g*_ = 0.14. Fit to *S*(*f*) = *A*/*f*^*α*^ with *α* = 0.6 ± 0.2 and *A* = 0.8 ± 1 × 10^−6^ (95% CI).

**Extended Data Fig. 8:**
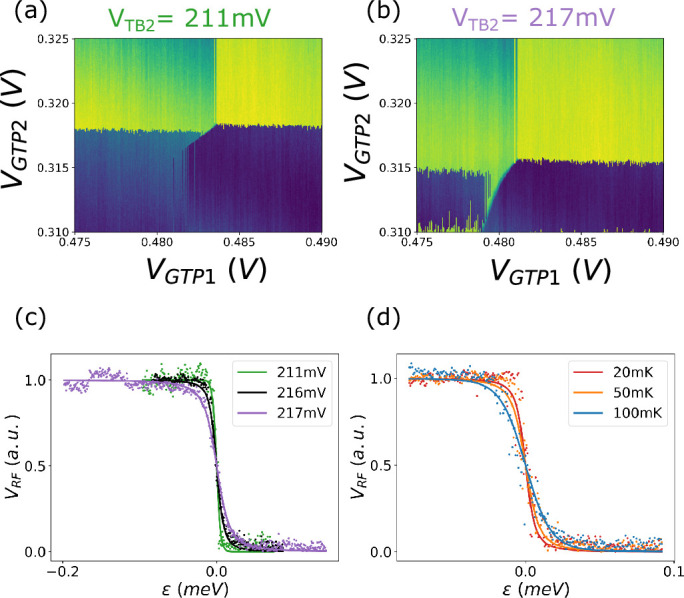
Tunnel coupling extraction. (**a**-**b**) ICT between (0,2) and (1,1) at two different barrier gate voltage. (**c**-**d**) Charge transition across the ICT with fits to Eq. 15 as a function of barrier gate voltages (**c**) and MXC temperature (**d**).

## Data Availability

The data supporting this work are available at Zenodo (10.5281/zenodo.19559265)^58^.

## Extended data

**Extended Data Table 1:** Overview of spin readout performance using Pauli Spin Blockade across platforms and sensors

Sensor	Platform	F_m (%)	t_read	Gamma_inverse	Reference
DC-SET	Si-MOS	99.3	150 μs	15 ms	Harvey-Collard2018
DC-SET	Si-MOS	99.95*	100 μs	undisclosed	Steinacker2024a
RF-SET	Si/SiGe	99.9	2 μs	18.2 ms	Takeda2024
RF-SET	Si/SiGe	82.9	2.1 μs	11 μs	Connors2020
RF-SET	Si-MOS	97.8	50 μs	10 ms	Huang2024
RF-SET	Si-MOS	99.96*	100 μs	undisclosed	Steinacker2024b
SEB	Si/SiGe	99.2	100 μs	1.03 ms	Borjans2021
SEB	Si-NW	99.9	20 μs	32 ms	Niegemann2022
SEB	Si-NW	99.2	6 μs	230 μs	Oakes2023
SEB	Si-MOS	99.92	340 μs	74 ms	This work
in-situ	Si/SiGe	98	6 μs	159 μs	Zheng2019
in-situ	Si-NW	98	0.5 μs	0.91 ms	Urdampilleta2019
in-situ	Si-MOS	73	2.6 ms	4.5 ms	West2019
in-situ	Si-MOS	70	8 μs	24 μs	Chittock-Wood2026

The results of this work are in bold. This table does not included latched readout in as it requires a nearby reservoir and therefore limits its scalability. *Fidelity calculated from SPAM using gate set tomography.

**Extended Data Table 2:** Readout resonator parameters

Parameter	2DEG off	2DEG on
f_0 (MHz)	583.9	578.6
Q_r	104	74.8
Q_int	197	106
β	0.9	0.42
C_p (pF)	0.55	0.56
R_C (kΩ)	72.1	38.6

Obtained from Eqs.~(3-8) calculated on data of Extended Data Fig.~2.
