# Supplementary material for: High-fidelity dispersive spin sensing in a tunable unit cell of silicon MOS quantum dots
Source: Nat Sens. 2026 Jul 7;1(7):617–26. doi: 10.1038/s44460-026-00084-6 (PMC13368588; doi:10.1038/s44460-026-00084-6)
Supplement: Supplementary file 1 — Supplementary discussion [file 44460_2026_84_MOESM1_ESM.pdf]

nature sensors

Supplementary information https://doi.org/10.1038/s44460-026-00084-6

High-fidelity dispersive spin sensing in a tunable unit cell of silicon MOS quantum dots

In the format provided by the authors and unedited

Supplementary Information

Supplementary Note A: Spin readout fidelity benchmark

We gather reported spin readout fidelities in semiconductor nanostructures from the literature in Fig. 1(a) and Table I. Our focus is on singlet-triplet and parity readout measured in single-shot via Pauli spin blockade (PSB) and we report best fidelity F_{m}^{*} calculated using the threshold method or by SPAM measurement at optimal readout time t_{read}. We consider different semiconductor platforms such as the heterostructure Si/SiGe, silicon nanowire (NW) and silicon MOS planar. While both silicon structures are compatible with foundry-like fabrication, the latter can more readily accommodate two-dimensional qubit layout (such as those in Fig. 6 of the main text). We also compare readout using different charge sensing mechanisms including the DC and RF-SET, the SEB and in-situ gate based readout. In-situ (or gate-based) is the most versatile readout but its fidelity is limited in planar devices as we explain below. SEBs take up some space in the QPU but can be compatible with large scale $2 \times N$ array of qubits as discussed in the main text, making them an attractive sensor.

The comparison with the state-of-the-art highlights that this first demonstration of a SEB in a Si planar device is already able to reach fidelity and readout times similar to SETs. The SEB also performs better than in-situ readout in Si-MOS, which can be attributed to the favourable triplet relaxation times. We also note that higher speed has been achieved for dispersive sensing in other platforms, due to higher lever-arm for NW and lower charge noise for Si/SiGe devices. The impact of these on readout fidelity is visible on the SNR in Eq. 2 of the main text via lever arm α and in Eq. 14 via the internal Q-factor Q_{int} representing internal resonator losses. This provides ways to reduce the minimum integration time τ_{min} (i.e. to increase its SNR) which effectively improve spin readout speed, as we show in Fig. 1(b). In silicon planar devices, while the gate lever-arm is limited by fabrication and often beyond control, resonator optimisation can still be improved to provide better impedance matching $\beta = 1$ and lower internal losses Q_{int}. Finally, quantum-limited parametric amplification could be utilised to further reduce τ_{min} of the SEB [1].

Supplementary Note B: Experimental set-up and resonator characterisation

The device used is a 300 mm wafer-scale MOS planar structure fabricated at the industrial-grade IMEC facility [14]. Three layers of overlapping PolySi gates are defined above an unpatterned ^{nat}Si substrate, providing the electrostatic potential to form QDs. Electrons are loaded from a nearby 2D-electron gas extended via accumulation gates from localised reservoirs of dopants. The chip is placed on a Printed Circuit Board (PCB) made of Rogers 4003C material and gold finish, which hosts a tank circuit for reflectometry.

The LC resonator circuit used for readout comprises a NbTiN spiral inductor $L = 88.7$ nH and a surface mount coupling capacitor $C_c = 0.2$ pF and is shown in Fig.2(a). An RF signal is sent to the device after 30 dB of attenuation at different stages of the dilution refrigerator, passband RF filtering with Mini-Circuits VLF-1800+ VLF145+ and a directional coupler Mini-Circuit ZFDC-20-33-S+ at the cold plate stage, adding 12 dB attenuation. The reflected

Sensor	Platform	F_{m}^{*}	t_{read}	Γ^{-1}	Reference
DC-SET	Si-MOS	99.3	150 µs	15 ms	Harvey-Collard2018 [2]
DC-SET	Si-MOS	99.95†	100 µs	undisclosed	Steinacker2024a [3]
RF-SET	Si/SiGe	99.9	2 µs	18.2 ms	Takeda2024 [4]
RF-SET	Si/SiGe	82.9	2.1 µs	11 µs	Connors2020 [5]
RF-SET	Si-MOS	97.8	50 µs	10 ms	Huang2024 [6]
RF-SET	Si-MOS	99.96†	100 µs	undisclosed	Steinacker2024b [7]
SEB	Si/SiGe	99.2	100 µs	1.03 ms	Borjans2021 [8]
SEB	Si-NW	99.9	20 µs	32 ms	Niegemann2022 [9]
SEB	Si-NW	99.2	6 µs	230 µs	Oakes2023 [8]
**SEB**	**Si-MOS**	**99.92**	**340 µs**	**74 ms**	**This work**
in-situ	Si/SiGe	98	6 µs	159 µs	Zheng2019 [10]
in-situ	Si-NW	98	0.5 µs	0.91 ms	Urdampilleta2019 [11]
in-situ	Si-MOS	73	2.6 ms	4.5 ms	West2019 [12]
in-situ	Si-MOS	70	8 µs	24 µs	Chittock-Wood2026 [13]

TABLE I: (a) Overview of spin readout performance using Pauli Spin Blockade across platforms and sensors. The results of this work are in bold. This table does not included latched readout in as it requires a nearby reservoir and therefore limits its scalability. †:Fidelity calculated from SPAM using gate set tomography.

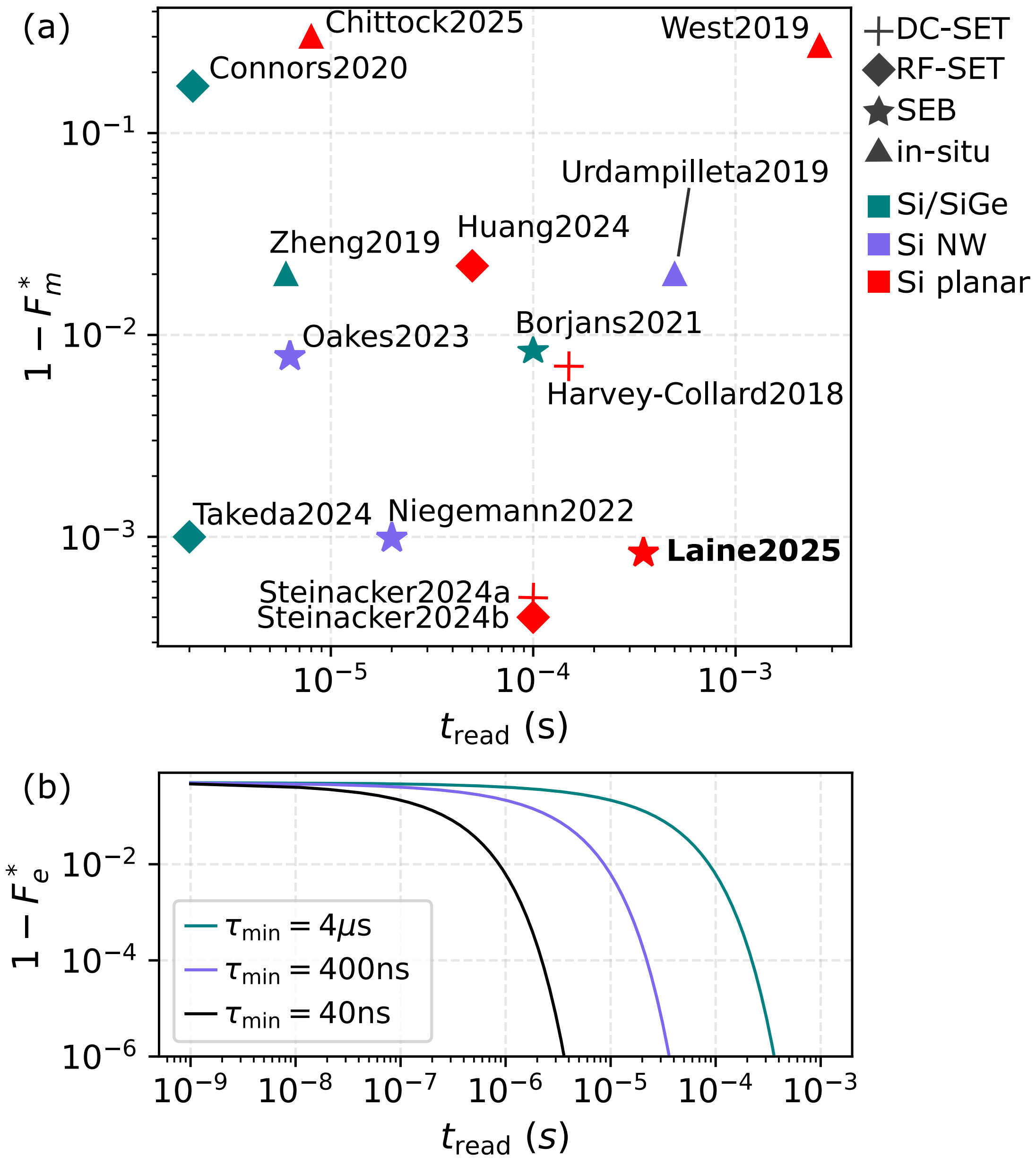

FIG. 1: Spin readout fidelity in the literature and possible improvement. (a) Spin readout fidelity using Pauli Spin Blockade across platforms and sensors. The data and references can be found in Table I. (b) Electrical fidelity plotted from Eq. 12 with voltage SNR taken as SNR $= \sqrt{t_{\mathrm{read}}/\tau_{\mathrm{min}}}$. The spin τ_{min} for this work is estimated to $\eta^{-1} \times \tau_{\mathrm{min}} \sim 4.1\mu s$ (Appendix I) which incorporates the loss of visibility η^{-1} due to distinguishing $(0,2)$ and $(1,1)$ Coulomb peaks when spin sensing. This shows that reducing our current SEB τ_{min} by an order of magnitude would allow to reach readout fidelity of 99% under 10µs.

signal is amplified at the 4K stage using a LNF-LNC0.2-3B ultra-low noise cryogenic amplifier and at room temperature with two Mini-Circuit ZX60-112LN. The device is operated in a Proteox MX Oxford Instruments dilution refrigerator at base temperature $T_{\mathrm{MXC}} = 10$mK.

To characterise the resonator, the reflected signal Γ is analysed by a Vector Network Analyser as shown in magnitude and phase in Fig. 2(b-c). We measure Γ as all the device gates are at zero voltage (black line) and when the 2DEG is extended under the SEB reservoir gate (red line). The shift of the resonance in phase and magnitude is due to changes in the capacitance and resistance from the formation of the 2DEG. After removing the background from fitting the standing wave, we fit the resonance to a Gaussian to find the resonant frequency f_0, and the full-width half maximum

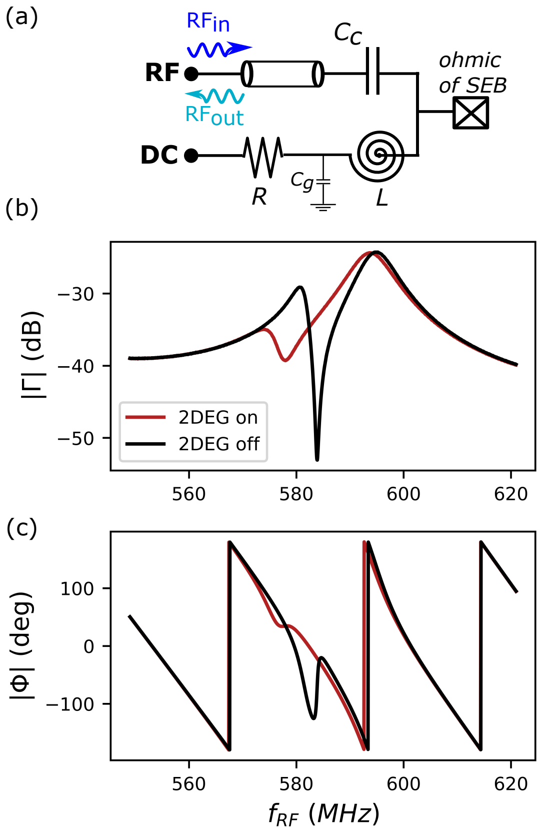

FIG. 2: Readout resonator and its characterisation. (a) Parallel LC circuit used for reflectometry. (b) VNA traces taken at 10mK, when bottom reservoir gate is at to turn-on voltage point and electron 2DEG is accumulated (red line) and when all gate voltages are at zero (black line).

	2DEG off	2DEG on
f_0 (MHz)	583.9	578.6
Q_r	104	74.8
Q_int	197	106
β	0.90	0.42
C_p (pF)	0.55	0.56
R_C (kΩ)	72.1	38.6

TABLE II: Readout resonator parameters. Obtained from Eqs (3-8) calculated on data of Fig. 2.

(FWHM) Δf. We then extract the coupling β and quality factor Q of resonator as:

$$\beta = \frac{\Gamma_V(f_0) - 1}{\Gamma_V(f_0) + 1}, \quad (1)$$

$$Q_\mathrm{r} = f_0/\Delta f, \quad (2)$$

$$Q_\mathrm{int} = (1+\beta)Q_r, \quad (3)$$

$$(4)$$

and calculate the parasitic capacitance and equivalent resistance to ground as:

$$C_p = 1/(4\pi^2 f_0^2 L) - C_c \quad (5)$$

$$R_C = Q_\mathrm{int}\sqrt{\frac{L}{C_c + C_p}} \quad (6)$$

These terms are gathered in Table II for both 2DEG off and on.

For charge sensing, the SEB dot is operated in the many-electron regime (see Supplementary Note D), with its ohmic contact connected to the LC resonator. When the electrostatic potential of the SEB dot and the reservoir are near alignment, the RF signal induces an electron to cyclically tunnel between them, contributing to the measured electrical impedance of the SEB. Changes in this impedance, caused, for example, by changes in the local potential of the SEB, are detected in the reflected RF signal which is demodulated with a DC1670A I/Q demodulator, low-pass filtered by SR560 Stanford voltage pre-amplifiers with a cut-off frequency of either 10 kHz or 1 MHz, and acquired with an M4i digitiser. The change in signal in the (I, Q) plane is then projected onto the axis that maximises the response and renormalised as $\Delta V_{\rm rf}/V_0$ where V_0 is the maximum voltage change. In the vicinity of a Coulomb blockade peak, the SEB is highly sensitive to its electrostatic environment. Due to their mutual capacitive coupling, changes in the charge configuration of the DQD can be detected by shifts in the SEB signal.

RF frequency and power are optimised to enhance the signal contrast $\delta V_{\rm RF}$ - as introduced in the main text. The RF frequency is optimised at $f_0 = 576$ MHz and power to $P_{\rm in} = -80$ dB to the device.

Supplementary Note C: Spin preparation and readout

To prepare the appropriate mixture of singlet and triplet states for PSB, we exploit the $S - T_-$ anticrossing and the $S - T_0$ dephasing in the (1,1) region. We first wait in the (0,2) region (point 'I' in Fig. 2(a-b) of the main text) for 10 times the spin relaxation time so that the system is relaxed in the $|(0,2)S\rangle$ state.

Next, we pulse into the (1,1) region (point 'P'). Depending on the ramp rate used to traverse the $S - T_-$ avoided crossing, either the $|\mathrm{S}(1,1)\rangle$ or $|\mathrm{T}_-(1,1)\rangle$ state will be populated. To calibrate the avoided crossing, we vary the ramp rate and measure the probability of initialising $|\mathrm{T}_-(1,1)\rangle$, as shown in Fig. 3. On this plot, we have converted the gate voltage ramp rate to eV/s using lever arm $\alpha = 0.14$ from Supplementary Note and the y-axis is normalised such that high voltage ($\Delta V_{\rm rf}/V_0 = 1$) corresponds to blockaded signal i.e. triplets, and low voltage ($\Delta V_{\rm rf}/V_0 = 0$) corresponds to singlets. The step shape of the curve is expected from the probability of a LZ transition given by

$$P_{LZ} = \exp\left(\frac{-2\pi\Delta^2_{S-T_-}}{\hbar v_{in}}\right), \tag{7}$$

which we fit to the data to extract $\Delta_{S-T_-} = 46.9 \pm 0.9$ neV. For state preparation, we fix the ramp rate such that it ensures approximately 50% of the population reaches $|(1,1)T_-\rangle$.

Once at point 'P', $|(1,1)S\rangle$ dephases into a mixture of $|(1,1)T_0\rangle$ and $|(1,1)S\rangle$. We characterise the dephasing time by varying the time t_P at detuning point 'P' in the (1,1) region. At this point, a combination of the spin-orbit interaction at the Si:SiO2 interface and the exchange interaction drives $|\uparrow\downarrow\rangle$ and $|\downarrow\uparrow\rangle$ mixing [13, 15]. We measure Rabi oscillations between the $|\mathrm{S}_0\rangle$ and $|\mathrm{T}_0\rangle$ states as shown in Fig. 3. We fit the oscillation to a damped sine wave:

$$V_{RF}(t) = A\exp(-t/T_2^*)\sin(f_{Rabi}t + \phi) \tag{8}$$

where A is a normalisation factor, $T_2^* = 0.4 \pm 0.1$ μs is the dephasing time, f_{Rabi} is the frequency of the oscillation and ϕ the phase. Waiting for 1 μs, this produces a mixture of states with expected probabilities $p(|\mathrm{S}\rangle) \simeq 0.25$, $p(|\mathrm{T}_0\rangle) \simeq 0.25$ and $p(|\mathrm{T}_-\rangle) \simeq 0.5$.

When performing readout, the tunnelling transition from (1,1) to (0,2) is not observed as it occurs on a timescale much faster than the sensor's minimum integration time, $\tau_{\rm min}$, (defined as the integration time required to distinguish (1,1) from (0,2) with SNR of one), which we measure as $\tau_{\rm min} = 3.3$ μs (see Supplementary Note J). Given the charge tunnelling process is fast, the relaxation rate Γ_{T_-} is primarily limited by the spin-flip rate, and Γ_{T_0} by spin dephasing. We therefore expect $\Gamma_{\mathrm{T}_-} \ll \Gamma_{\mathrm{T}_0}$ as spin flips are driven by weaker interactions, such as spin-orbit coupling [16, 17]. Hence, we assign the shorter of the measured relaxation times to $\Gamma^{-1}_{\mathrm{T}_0} = 170 \pm 2$ μs, and the longer to $\Gamma^{-1}_{\mathrm{T}_-} = 290 \pm 3$ ms in the regime of $V_{\rm GTB2} = 212$ mV.

We note that throughout these measurements, barrier gate TB2 is held constant during a given pulse sequence (i.e. during readout and initialisation). To minimise the effect of different GTB2 potentials on the preparation of initial states, we calibrate the ramp and control time for each $V_{\rm GTB2}$.

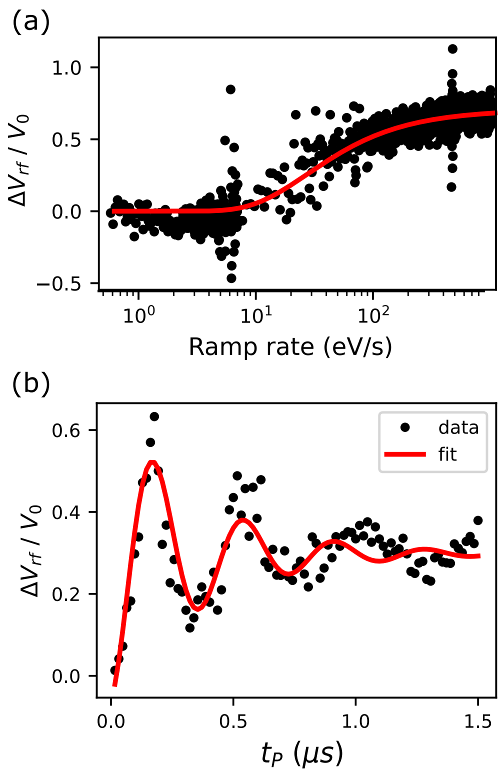

FIG. 3: Characterisation of the qubit dots. (a) LZ single passage experiment, where ramp rate is varied across the Δ_{S-T_-} anticrossing. Fit to Eq. 7 with $\Delta_{S-T_-} = 46.9 \pm 0.9$ neV. (b) Measurement of dephasing time from coherent oscillation measured in the singlet-triplet basis. The oscillation is fitted to a damp sine wave with $T_2 = 0.4 \pm 0.1$ µs, $f_{Rabi} = 17.0 \pm 0.4$ MHz and $A = 0.35 \pm 0.04$ fitted to Eq. 8.

Supplementary Note D: Characterisation and tuning of the SEB dot

We use Coulomb peak thermometry to find the SEB lever arm and electron temperature. We use a SEB DRT peak that is nor power or lifetime broadened. We fit the FWHM of the peak to [18]

$$\mathrm{FWHM} = \frac{3.53\mathrm{k_B}}{\mathrm{e}\alpha}\sqrt{\mathrm{T}_{\mathrm{MXC}}^2 + \mathrm{T}_{\mathrm{e}}^2} \tag{9}$$

where α is the gate lever arm, $\mathrm{T_{MXC}}$ is the mixing chamber temperature and $\mathrm{T_e}$ is the electron temperature. Fig. 4(a) shows the fit where we extract $\mathrm{T_e} = 90 \pm 11$ mK and $\alpha = 0.17 \pm 0.01$.

To tune the SEB, a dot is formed in the many-electrons regime under the plunger gate BP1 by changing the barrier gate BB1. This is shown in the stability map of Fig. 4(b) which displays several DRT in the power broadened regime e.g. when $P_{\mathrm{in}} = -70$ dB is sent to the device. For a barrier gate voltage in the range 0.2—0.3 V, the first electron is loaded into the SEB dot from voltage $V_{\mathrm{GBP1}} \simeq 0.5$ V. Given the visible loading voltage of $\Delta V \simeq 33$ mV, we deduce an approximate electron number of 15—21 from the stability map. We notice a regime in which the peak is broadened and the signal reduced which is the lifetime broadened regime. Beyond this, a larger BB1 voltage causes the formation of an additional dot under BB1, which couples strongly to the SEB dot, as hinted by the change in slope above 0.3 V in barrier gate voltage.

To operate the SEB as a charge sensor, we optimise the power sent to the device such that the peak linewidth is reduced and therefore η increased without reduction in peak height, resulting in $P_{\mathrm{in}} = -80$ dB. In this regime, the best SNR is obtained by fine tuning gate BB1 in Fig. 3(c), as detailed in the main text.

Supplementary Note E: Data Pre-Processing for Readout

In this section, we address the issue of sensor drift, which degrades readout fidelity. To mitigate this problem, we acquire data during the final 120 µs of the plunge (‘P’) phase in the voltage sequence when the system is in the known

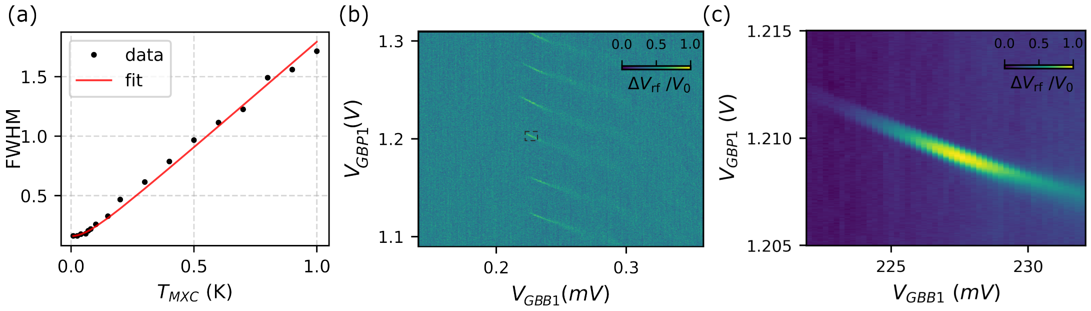

FIG. 4: Characterisation of the SEB dot. (a) SEB thermometry. Measurement of the SEB FWHM as a function of temperature and fit to Eq. 9 to extract $T_e = 90 \pm 11$ mK and $\alpha = 0.17 \pm 0.01$. (b) Stability map showing six DRT where the lowest electron number ($V_{\text{GBP1}} < 1.1$ V) is 15 . The dashed rectangle corresponds to the range used for SNR optimisation in Fig. 3. (c) Zoom on the operating region where the SNR is optimised as in Fig. 3(b). Notice that the stability map of Fig. 3(a) corresponds to a different thermal cycle and is obtained at lower power, which explains some voltage shifts.

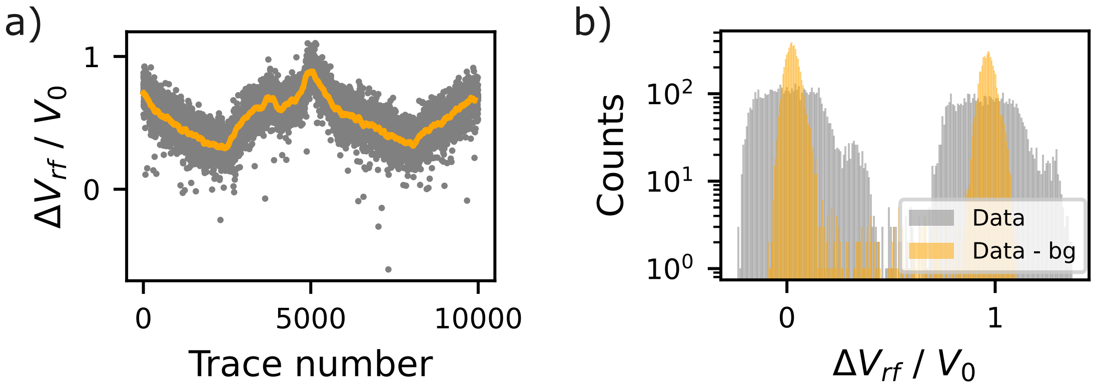

FIG. 5: Data Pre-Processing for Readout. (a) Sensor drift observed during the readout fidelity process (shown in grey), whose fluctuations are mitigated by applying a moving average filter with a window size of 50, as shown in orange. The interval between consecutive readout traces depends on the duration of the entire readout sequence, which includes an initialization phase requiring a wait time of approximately five times T_1. This example corresponds to the longest T_1, resulting in the longest readout sequence, lasting nearly one second. (b) Histograms comparing 10,000 single-shot traces averaged over $t_{\text{int}} = 204$ μs before (grey) and after (orange) background effect removal.

(1,1) charge configuration. This allows capturing the background noise before actual data collection begins. However, the background signal is susceptible to noise, as shown by the grey data points in Fig. 5(a). To reduce this noise and stabilise the readout, we average the background from the last 50 readout traces. This averaged value is subtracted from each readout trace to correct for the sensor drift.

Importantly, we only use background measurements of traces taken previously (in the past), ensuring that this approach remains compatible with real-time quantum algorithm operations. This is crucial because, during the execution of a quantum algorithm, decisions and corrections must rely on data that has already been collected; future measurements are not available in real time.

The effectiveness of this method is demonstrated in Fig. 5(a), where the orange line represents the averaged background over time. After applying the correction, the sensor signal no longer drifts, resulting in a more stable output. Further evidence is shown in Fig. 5(b), where the histogram of averaged single-shot traces over the first 204 µs reveals a reduced overlap between peaks after drift removal, indicating enhanced signal discrimination.

This method is compatible with quantum algorithms, as periodic background measurements could be performed during quantum operations, provided that the sensor-rf signal does not significantly affect qubit gate performance. Continuously tracking and compensating for sensor drift ensures reliable and high-fidelity readout throughout dynamic quantum computations.

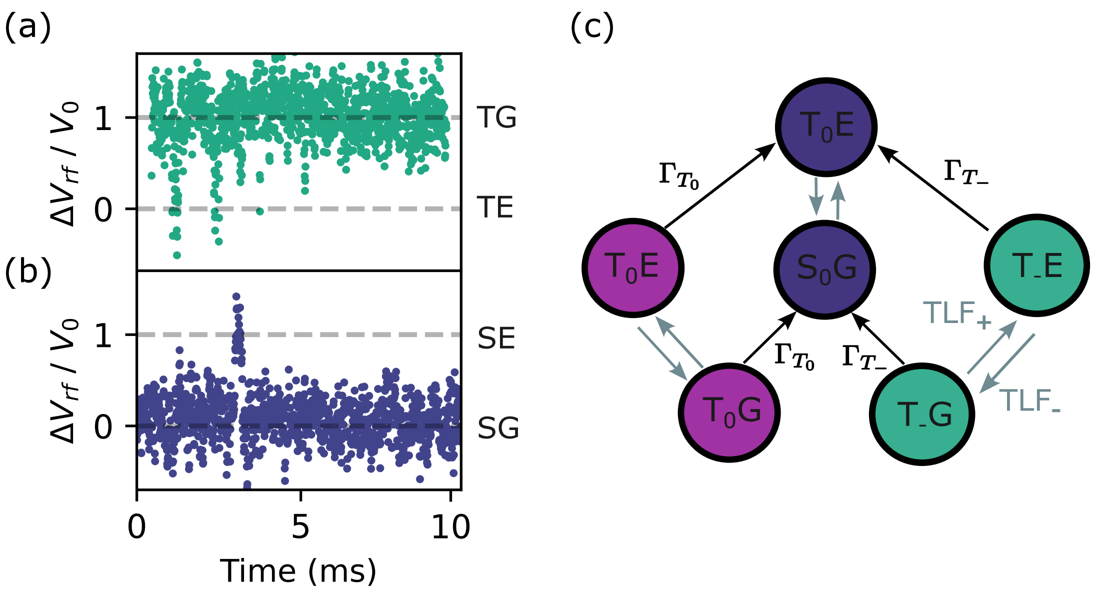

FIG. 6: TLF effect on Sensor Signal. (a) Effect of the two-level fluctuator (TLF) on the sensor signal for the singlet state ($|\mathrm{S}\rangle$). When the TLF is in the ground state (G), the sensor signal is centred at $\Delta V_{\mathrm{rf}}/V_0 = 0$. However, when the TLF switches to the excited state (E), the signal shifts to resemble a triplet state ($\Delta V_{\mathrm{rf}}/V_0 = 1$). (b) Effect of the two-level fluctuator (TLF) on the sensor signal for the triplet states ($|\mathrm{T}_-\rangle$). (c) Markov chain representing the six hidden states, including the transitions of the two-level fluctuator: TLF_+ for transitions from the ground (G) to the excited (E) state, and TLF_- for transitions from the excited (E) to the ground (G) state.

Supplementary Note F: Data Simulation with a HMM

Hidden Markov Models (HMMs) describe stochastic processes that switch between discrete states, with transitions governed by a probability matrix. They are well suited to physical systems such as tunneling or relaxation events, where dynamics are memoryless. Each state produces a signal with its own mean and variance, which allows the model to capture realistic noise and fluctuations. Within this framework, different noise sources—Gaussian, charge, or correlated—can be included naturally.

Here we use an HMM to simulate and classify three spin states, $|\mathrm{S}\rangle$, $|\mathrm{T}_-\rangle$, and $|\mathrm{T}_0\rangle$. The triplet states share the same signal level but differ in their transition dynamics. Analysis of single-shot traces shows that the signal is affected by Gaussian noise and by a two-level fluctuator (TLF) (see Fig. 6)

We first simulate the data using a simple HMM that excludes the TLF. This model has three hidden states. The triplet states, ($|\mathrm{T}_0\rangle$ and $|\mathrm{T}_-\rangle$), relax to the singlet state $|\mathrm{S}\rangle$ at their measured relaxation rates, which are extracted from double-exponential fits to ensemble-averaged traces. Each hidden state emits a constant signal with Gaussian noise, representing the sensor output for the charge states (1,1) and (0,2). The noise level is taken as the same across all states.

This simple HMM has five free parameters: the average signals $\mu_{(1,1)}$ and $\mu_{(2,0)}$, the Gaussian noise σ, and the decay rates Γ_{T_-} and Γ_{T_0}. These parameters are obtained from fits to the experimental data and refined using the Expectation–Maximization algorithm [19], which iteratively adjusts the initial state, transition, and emission probabilities to maximise the likelihood of reproducing the data.

While this model reproduces the basic signal statistics, it underfits the experimental data and fails to capture the full noise behaviour (see Fig. 7(b)). To address this, we include a TLF in a more refined HMM.

1. TLF Effect on Sensor Signal

TLFs are a major issue in $\mathrm{Si/SiO_2}$ devices where charge fluctuators are present at the interface and couple electrically to the qubit charge states, and the exact amount can vary from device to device. We model this by noticing that the TLF alters the sensor's output depending on its state. For $|\mathrm{S}\rangle$, the sensor emits a signal centred at $\Delta \mathrm{V_{rf}/V_0} = 0$ when the TLF is in the ground state (G). However, when the TLF switches to the excited state (E), the signal shifts dramatically to resemble a triplet signal ($\Delta \mathrm{V_{rf}/V_0} = 1$), as illustrated in Fig. 6(a).

A similar phenomenon occurs for $|\mathrm{T}_-\rangle$ and $|\mathrm{T}_0\rangle$, where the signals, typically centred at $\Delta\mathrm{V}_{\mathrm{rf}}/\mathrm{V}_0 = 1$ in the ground TLF state, shift to resemble a singlet signal ($\Delta\mathrm{V}_{\mathrm{rf}}/\mathrm{V}_0 = 0$) when the TLF is excited (Fig. 6(b)).

2. Extended HMM with TLF States

To account for this effect, the HMM is expanded to include six states: $|\mathrm{S}\rangle$G, $|\mathrm{S}\rangle$E, $|\mathrm{T}_-\rangle$G, $|\mathrm{T}_-\rangle$E, $|\mathrm{T}_0\rangle$G, and $|\mathrm{T}_0\rangle$E. These states incorporate both spin and TLF dynamics (see Fig. 6(c)). Transitions follow two mechanisms:

- **Spin State Transitions:** Triplet states ($|\mathrm{T}_-\rangle$, $|\mathrm{T}_0\rangle$) decay to $|\mathrm{S}\rangle$, preserving the TLF state. For example, $|\mathrm{T}_0\rangle$G decays to $|\mathrm{S}\rangle$G, and $|\mathrm{T}_0\rangle$E decays to $|\mathrm{S}\rangle$E.
- **TLF Switching:** Any state can transition between its G and E variants.

This extended HMM adds only two parameters, TLF+ and TLF−, which describe the transitions between the ground (G) and excited (E) states of the fluctuator. Despite this minimal increase in complexity, the model captures the system dynamics and noise far more accurately, closely matching the experimental data (see Fig. 7(b)).

Supplementary Note G: Single-shot SNR optimisation

For reflectometry readout, the voltage SNR can be given as [20]

$$\mathrm{SNR} = |\Delta\Gamma_{\mathrm{c.s.}}|\frac{\mathrm{V_{in}}}{\mathrm{V_n}}, \tag{10}$$

where $V_{in(n)}$ are the input RF and noise voltages, respectively and $\Delta\Gamma_{c.s.}$ is the change of signal due to a charge sensing event. For the SEB a DRT transition will cause a change of quantum capacitance given by Eq. 2. In the regime where this change is small ($Q_r\Delta C_{DRT}/C_{tot} << 1$) it causes a change of signal [21] :

$$\Delta\Gamma = \Delta C_{\mathrm{DRT}}\left[\frac{\partial\Gamma}{\partial C_{\mathrm{DRT}}}\right]_{f=f_{\mathrm{res}}} \tag{11}$$

The second term of the equation can be calculated as a function of the resonant frequency and therefore the tank circuit parameters such that :

$$\Delta\Gamma = i\frac{2\beta}{(1+\beta)^2}Q_{int}\frac{\Delta C_{\mathrm{DRT}}}{C_{\mathrm{tot}}} \tag{12}$$

where C_{tot} is the total capacitance of the system (including the SEB and matching network), and ΔC_{DRT} is the change in capacitance of the SEB as defined in the main text. Finally, we have to account that the signal measured for charge sensing does not correspond to the full height of the SEB peak. This is because we aim at differentiating peaks $(0,2)$ and $(1,1)$ and what we therefore measure is the signal contrast ΔV_{RF} as detailed in the main text and shown in Fig. 1(c). To account for this reduction in signal we introduce the parameter η such that:

$$\Delta\Gamma_{\mathrm{c.s.}} = \eta\Delta\Gamma, \tag{13}$$

where $\Delta\Gamma_{\mathrm{c.s.}}$ is the change in signal due to a charge sensing event. Combining Eqs.10, 12, 13 yield

$$\mathrm{SNR} = \eta\frac{2\beta}{(1+\beta)^2}\mathrm{Q_{int}}\frac{\Delta\mathrm{C_{DRT}}}{\mathrm{C_{tot}}}\frac{\mathrm{V_{in}}}{\mathrm{V_n}}. \tag{14}$$

Therefore, we see that maximising the SNR entails the optimisation of 1) the input amplitude or power via Eq. 10 2) the tank circuit parameters via Eq. 12 and 3) the DRT transition via Eq. 2. Power is optimised by increasing it until the SEB charge sensor peak gets broadened leading to a reduction of ΔC_D that dominates the SNR. We mitigate the back-action of the RF on relaxation times by tuning the SEB such that it is on resonance (peak) when the qubits are in the (0,2) ground state. The optimal power used is P_{in} = -80 dB. The tank circuit components are cleverly chosen as explained in Methods. Optimisation of the DRT is the focus of the main text.

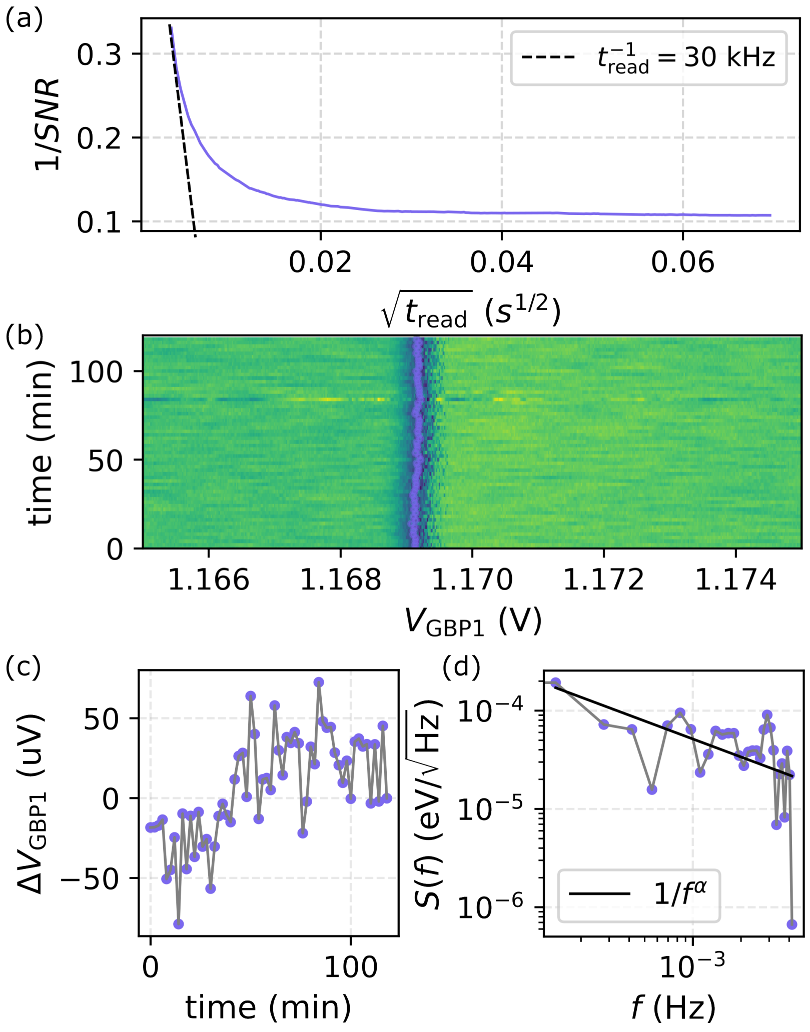

FIG. 7: Noise analysis. (a) Fast noise: at fixed signal 1/SNR $= \sigma$ is suppressed linearly with $1/\sqrt{t_{\text{read}}}$ up until it plateau for $t_{\text{read}}^{-1} \sim 30$kHz. This is where non-white noise such as charge noise starts to dominate. (b) Stability of the SEB: peak tracking of the SEB point with centre obtained from fit to $\cosh^{-2}$. (c) Slow noise : noise spectral density calculated from welch method and using gate lever-arm $\alpha_g = 0.14$. Fit to $S(f) = A/f^{\alpha}$ with $\alpha = 0.6 \pm 0.2$ and $A = 0.8 \pm 1 \times 10^{-6}$ (95% CI).

Supplementary Note H: Noise spectrum

We confirm the noise is dominated by white noise at short integration time by plotting 1/SNR with SNR obtained from Eq. 19 of the main text in the case of $\Gamma = 0$ (electrical fidelity). We use F_e^* measured for the best tuning point i.e. $V_{\text{GBB1}} = 227$ mV, and we show it in Fig. 7(a).

We also check the stability of the SEB tuning in time by tracking the SEB peak over two hours as shown in Fig. 7(b-c). This allows to extract the charge noise spectrum in the mHz regime which we fit to $S(f) = A/f^{\alpha}$ with $\alpha = 0.6 \pm 0.2$ and $A = 0.8 \pm 1 \times 10^{-6}$. These coefficients provide information on charge noise and are qualitatively compatible with the change of slope of Fig. 7(a) at longer timescale from $\alpha = 1$ to $\alpha < 1$. For device operation, we retune the SEB before each measurement sequence with an automated tuning function which finds the voltage point of maximum contrast as described in Fig. 2 of the main text. This allows to compensate for the slow drift visible in Fig. 7(c).

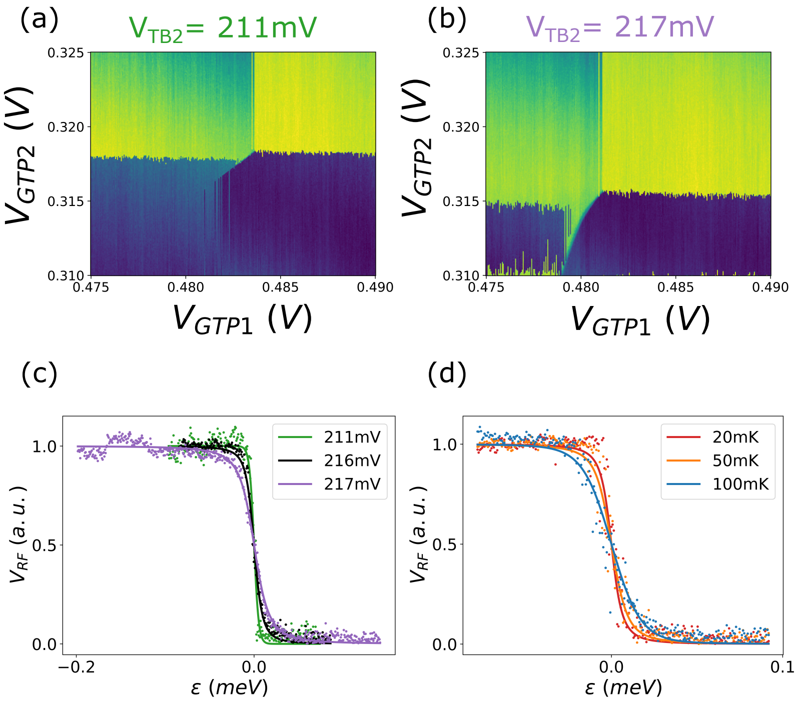

FIG. 8: Tunnel coupling extraction. (a-b) ICT between (0,2) and (1,1) at two different barrier gate voltage. (c-d) Charge transition across the ICT with fits to Eq. 15 as a function of barrier gate voltages (c) and MXC temperature (d).

Supplementary Note I: Tunnel coupling extraction

In Fig. 4 of the main text, we measure tunnel coupling value t_c by using the ICT linewidth and fitting it to [22]:

$$f(\epsilon) = \frac{1}{2}\left[1 - \frac{\epsilon}{\sqrt{\epsilon^2 + 4t_c^2}} \tanh\left(\frac{\sqrt{\epsilon^2 + 4t_c^2}}{2k_B T_e}\right)\right] \tag{15}$$

where ϵ is the detuning in eV and T_e the qubit electron temperature. The dependency of the ICT on tunnel coupling is visible in Fig. 8(a-b) where the ICT is shown for two different barrier gate voltages. Fits to Eq. 15 are shown in Fig. 8(c) which determine an upper bound for tunnel coupling value of $t_c = 8.0 \pm 0.5$ μeV in the regime where $V_{\mathrm{GTB2}} = 217$ mV. Values smaller than $t_c = 4.0 \pm 0.1$ μeV however are not accessible as the transition is temperature broadened at around $T_e = 40 \pm 10$ mK, also extracted from the fits obtained when varying the mixing chamber (MXC) temperature at a fixed barrier gate voltage as shown in Fig. 8(d). We notice the qubit electron temperature is lower than that of the SEB, which could be explained by power dissipation of the RF connected to the SEB.

Supplementary Note J: Minimum integration time

We projecting the RF signal on the principal component axis. The sensor is tuned such that the Coulomb peak is on resonance when the qubit is in the even parity state, meaning the signal in the (1,1) charge state approaches the top of the Coulomb peak.

To optimise the signal-to-noise ratio (SNR), we project the signal onto the axis of maximum visibility that connects the centre of the two circles. This axis is referred to as the ΔV_{RF} axis and is used throughout the text.

We can calculate the SNR of such a measurement as :

$$\mathrm{SNR} = \frac{\Delta V_{RF}}{\sigma_{RF}}, \tag{16}$$

where ΔV_{RF} is the distance between the centres of the two circles and σ_{RF} is the standard deviation of the signal distribution. Both parameters are obtained by fitting the histogram to a 2D bimodal Gaussian distribution.

Since the histogram reflects the spin signal, the SNR calculated here is for spin readout. However, we can also determine the SNR for the SEB charge sensor, which is slightly higher than the spin readout SNR. This difference arises because spin readout must differentiate between the (0,2) and (1,1) charge states, and the contrast between these states is smaller than the peak height, as seen in Fig. 1(c).

To account for this, we introduce the parameter η, which represents the fractional change in the signal due to a charge sensing event:$\mathrm{SNR}_{\mathrm{charge}} = \mathrm{SNR}_{\mathrm{spin}}/\eta$. With $\eta = 0.8$ calculated from Fig. 1(c) we measure an SNR of 10.0 ± 0.1 for charge readout using the SEB.

We then calculate the minimum integration time of the sensor τ_{min}, which is the integration time yielding a power SNR of 1. τ_{min} is given by [21]:

$$\tau_{\mathrm{min}} = t_{\mathrm{read}}/\mathrm{SNR}^2. \tag{17}$$

We find a minimum integration time for the SEB of $\tau_{\mathrm{min}} = 3.3 \pm 0.1\mu\mathrm{s}$.

[1] S. Schaal, I. Ahmed, J. A. Haigh, L. Hutin, B. Bertrand, S. Barraud, M. Vinet, C.-M. Lee, N. Stelmashenko, J. W. A. Robinson, J. Y. Qiu, S. Hacohen-Gourgy, I. Siddiqi, M. F. Gonzalez-Zalba, and J. J. L. Morton, Phys. Rev. Lett. **124**, 067701 (2020).

[2] P. Harvey-Collard, B. D'Anjou, M. Rudolph, N. T. Jacobson, J. Dominguez, G. A. Eyck, J. R. Wendt, T. Pluym, M. P. Lilly, W. A. Coish, M. Pioro-Ladrière, and M. S. Carroll, Physical Review X **8**, 10.1103/PhysRevX.8.021046 (2018).

[3] P. Steinacker, N. Dumoulin Stuyck, W. H. Lim, T. Tanttu, M. Feng, S. Serrano, A. Nickl, M. Candido, J. D. Cifuentes, E. Vahapoglu, S. K. Bartee, F. E. Hudson, K. W. Chan, S. Kubicek, J. Jussot, Y. Canvel, S. Beyne, Y. Shimura, R. Loo, C. Godfrin, B. Raes, S. Baudot, D. Wan, A. Laucht, C. H. Yang, A. Saraiva, C. C. Escott, K. De Greve, and A. S. Dzurak, Nature **646**, 81 (2025).

[4] K. Takeda, A. Noiri, T. Nakajima, L. C. Camenzind, T. Kobayashi, A. Sammak, G. Scappucci, and S. Tarucha, npj Quantum Information **10**, 22 (2024).

[5] E. J. Connors, J. J. Nelson, and J. M. Nichol, Physical Review Applied **13**, 10.1103/PhysRevApplied.13.024019 (2020).

[6] J. Y. Huang, R. Y. Su, W. H. Lim, M. Feng, B. van Straaten, B. Severin, W. Gilbert, N. Dumoulin Stuyck, T. Tanttu, S. Serrano, J. D. Cifuentes, I. Hansen, A. E. Seedhouse, E. Vahapoglu, R. C. C. Leon, N. V. Abrosimov, H.-J. Pohl, M. L. W. Thewalt, F. E. Hudson, C. C. Escott, N. Ares, S. D. Bartlett, A. Morello, A. Saraiva, A. Laucht, A. S. Dzurak, and C. H. Yang, Nature **627**, 772 (2024).

[7] P. Steinacker, T. Tanttu, W. H. Lim, N. D. Stuyck, M. Feng, S. Serrano, E. Vahapoglu, R. Y. Su, J. Y. Huang, C. Jones, *et al.*, Nature communications **16**, 3606 (2025).

[8] F. Borjans, X. Mi, and J. Petta, Phys. Rev. Appl. **15**, 044052 (2021).

[9] D. J. Niegemann, V. El-Homsy, B. Jadot, M. Nurizzo, B. Cardoso-Paz, E. Chanrion, M. Dartiailh, B. Klemt, V. Thiney, C. Bäuerle, P. A. Mortemousque, B. Bertrand, H. Niebojewski, M. Vinet, F. Balestro, T. Meunier, and M. Urdampilleta, PRX Quantum **3**, 10.1103/PRXQuantum.3.040335 (2022).

[10] G. Zheng, N. Samkharadze, M. L. Noordam, N. Kalhor, D. Brousse, A. Sammak, G. Scappucci, and L. M. Vandersypen, Nature Nanotechnology **14**, 742 (2019).

[11] M. Urdampilleta, D. J. Niegemann, E. Chanrion, B. Jadot, C. Spence, P. A. Mortemousque, C. Bäuerle, L. Hutin, B. Bertrand, S. Barraud, R. Maurand, M. Sanquer, X. Jehl, S. De Franceschi, M. Vinet, and T. Meunier, Nature Nanotechnology **14**, 737 (2019).

[12] A. West, B. Hensen, A. Jouan, T. Tanttu, C. H. Yang, A. Rossi, M. F. Gonzalez-Zalba, F. Hudson, A. Morello, D. J. Reilly, and A. S. Dzurak, Nature Nanotechnology **14**, 437 (2019).

[13] J. F. Chittock-Wood, R. C. C. Leon, M. A. Fogarty, T. Murphy, F.-E. von Horstig, S. M. Patomäki, G. A. Oakes, J. Williams, N. Johnson, J. Jussot, S. Kubicek, B. Govoreanu, D. F. Wise, J. J. L. Morton, and M. F. Gonzalez-Zalba, Nature Electronics **9**, 314 (2026).

[14] T. N. Camenzind, A. Elsayed, F. A. Mohiyaddin, R. Li, S. Kubicek, J. Jussot, P. Van Dorpe, B. Govoreanu, I. Radu, and D. M. Zumbühl, Materials for Quantum Technology **1**, 10.1088/2633-4356/AC40F4 (2021).

[15] R. M. Jock, N. T. Jacobson, P. Harvey-Collard, A. M. Mounce, V. Srinivasa, D. R. Ward, J. Anderson, R. Manginell, J. R. Wendt, M. Rudolph, T. Pluym, J. K. Gamble, A. D. Baczewski, W. M. Witzel, and M. S. Carroll, Nature Communications **9**, 10.1038/s41467-018-04200-0 (2018).

[16] T. Meunier, I. Vink, L. W. van Beveren, K. Tielrooij, R. Hanson, F. Koppens, H. Tranitz, W. Wegscheider, L. Kouwenhoven, and L. Vandersypen, Physical review letters **98**, 126601 (2007).

[17] K. Shen and M. Wu, Physical Review B **76**, 235313 (2007).

[18] I. Ahmed, A. Chatterjee, S. Barraud, J. J. Morton, J. A. Haigh, and M. F. Gonzalez-Zalba, Communications Physics **1**, 10.1038/s42005-018-0066-8 (2018).

[19] P. Chang, G. Harper-Donnelly, A. Kara, X. Li, S. Linderman, and K. Murphy, Dynamax: A library for probabilistic modeling with neural networks (2024), accessed: December 2024.

[20] F. Vigneau, F. Fedele, A. Chatterjee, D. Reilly, F. Kuemmeth, F. Gonzalez-Zalba, E. Laird, and N. Ares, Applied Physics Review **10**, 10.1063/5.0088229 (2023).

[21] V. Ciriano Tejel, *High-fidelity, compact readout of spins in silicon quantum dots*, Ph.D. thesis, UCL (University College London) (2022).

[22] J. R. Petta, A. C. Johnson, C. M. Marcus, M. P. Hanson, and A. C. Gossard, Physical Review Letters **93**, 10.1103/PhysRevLett.93.186802 (2004).
